# Supplementary material for: Yeast TLDc domain proteins regulate assembly state and subcellular localization of the V-ATPase
Source: EMBO J. 2024 Apr 8;43(9):9. doi: 10.1038/s44318-024-00097-2 (PMC11066047; doi:10.1038/s44318-024-00097-2)
Supplement: Supplementary file 1 — Appendix [file 44318_2024_97_MOESM1_ESM.pdf]

Appendix

Table of contents

Appendix Figure S1: Structural mapping of the detected cross-links onto the structure of the EGO, SEA and TORC1 complexes. 2

Appendix Figure S2: Repetitions of azido-myristate labeling and subcellular fractionation experiments from Figure 4 3

Appendix Figure S3: Repetition of the vacuole proteomics experiments of oxr1Δ and rtc5Δ strains. 4

Appendix Figure S4: Whole cell proteomics of oxr1Δ and rtc5Δ strains. 5

Appendix Figure S5: Vacuole proteomics of rav1Δ strains compared with rav1Δ strains overexpressing either Rtc5 or Oxr1. 6

Appendix Figure S6: Genetic interactions between yeast TLDc domain-containing proteins and isoforms of V-ATPase subunit a. 7

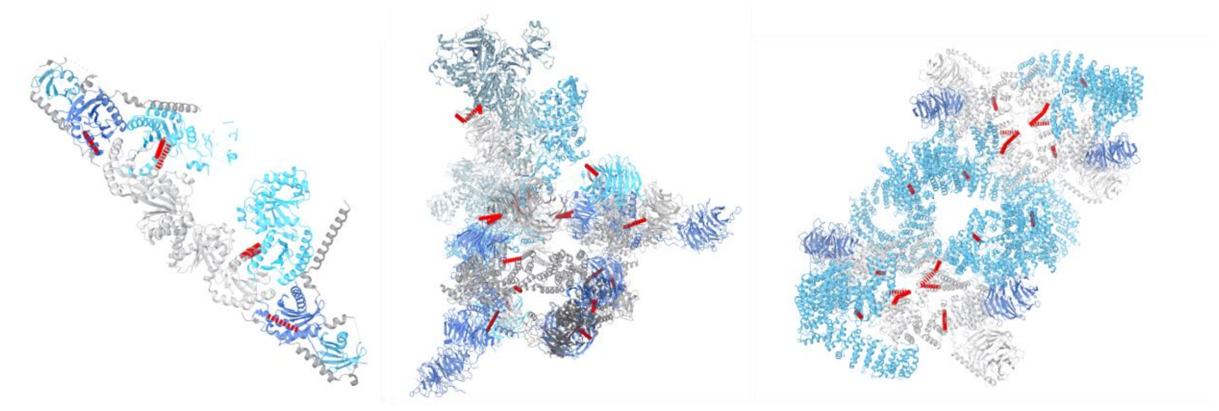

EGO complex (PDB ID: 6JWP) | SEA complex (PDB ID: 8ADL) | TORC1 complex (PDB ID: 7PQH)

Appendix Figure S1: Structural mapping of the detected cross-links onto the structure of the EGO, SEA and TORC1 complexes

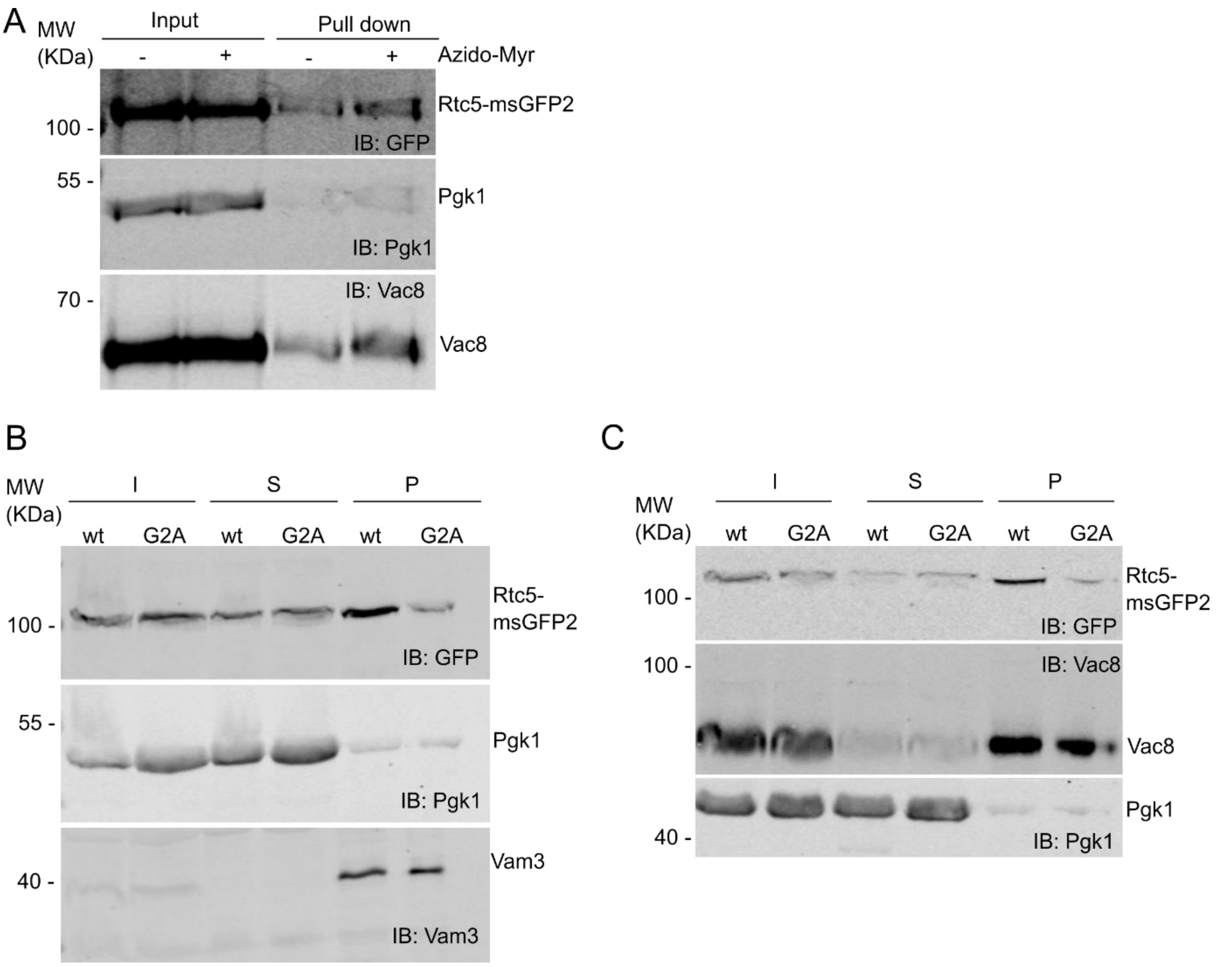

Appendix Figure S2: Repetitions of azido-myristate labeling and subcellular fractionation experiments from Figure 4

A) Cells expressing Rtc5-msGFP2 under the control of the strong constitutive *TEF1* promoter were labeled with azido-myristate (+) or mock-treated (-). A click chemistry-based conjugation of the azido myristate with alkyne biotin was performed in the lysates and myristoylated proteins were pulled down using a streptavidin matrix. Vac8 is shown as a positive control of a myristoylated protein and Pgk1 as a negative control. In each membrane, the protein recognized by the primary antibody used for the immunoblot (IB) is indicated. This is a repetition of the experiment shown in Figure 4 B.

B – C) Analysis of membrane association of Rtc5 and the Rtc5(G2A) mutant. A subcellular fractionation experiment was performed using lysates from strains expressing C-terminally msGFP2 tagged Rtc5 and the Rtc5(G2A) mutant. Pgk1 is shown as a cytosolic marker protein, Vac8 or Vam3 as membrane protein markers. The gels show an input corresponding to the lysate after a low-speed centrifugation to remove unbroken cells and nuclei (I), a supernatant sample (S) after centrifugation at 20000g for 20 minutes and the corresponding pellet sample (P), equivalent amounts of all samples were loaded. In each membrane, the protein recognized by the primary antibody used for the immunoblot is indicated (IB). These experiments are repetitions of the experiment shown in Figure 4 C.

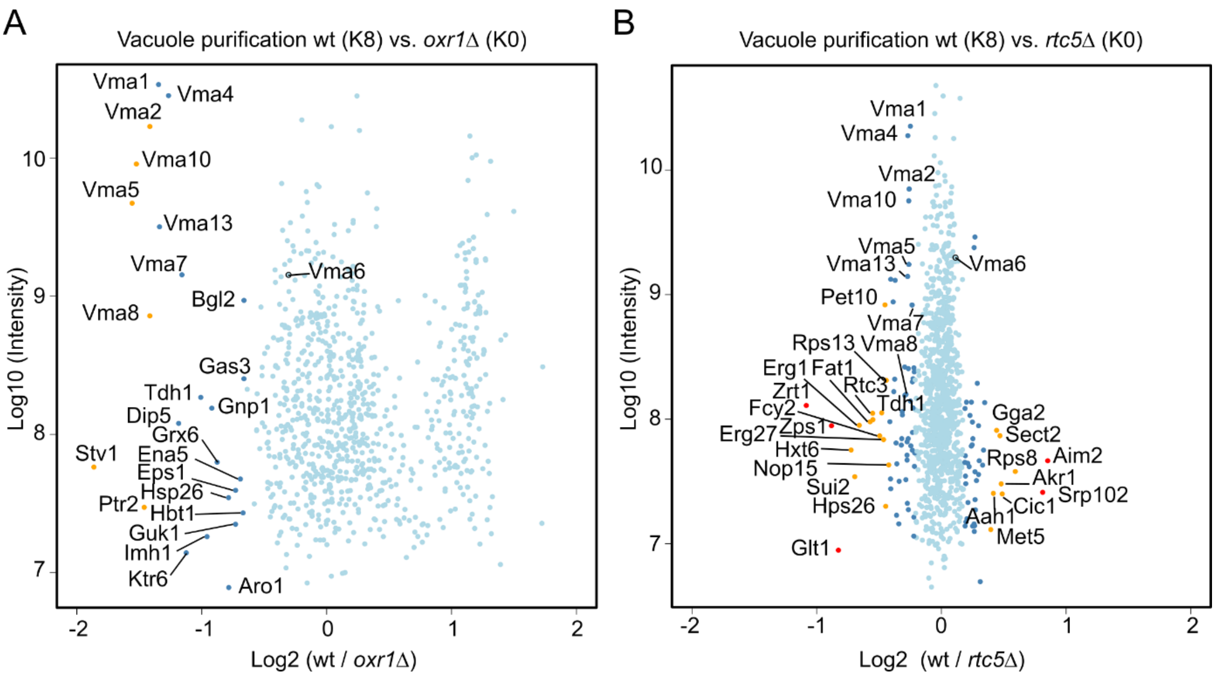

Appendix Figure S3: Repetition of the vacuole proteomics experiments of *oxr1*Δ and *rtc5*Δ strains

A and B) Same experiment as Figure 5 D and E, with switched heavy and light labeling. SILAC-based vacuole proteomics of cells lacking either *OXR1* (A) or *RTC5* (B) compared with the wt strain. Log10 of the detected protein intensities are plotted against Log2 of the heavy/light SILAC ratios. Significant outliers are color coded in red ($P < 1e{-}14$), orange ($P < 0.0001$), or dark blue ($P < 0.05$); other identified proteins are shown in light blue. Statistical comparison is based on a two-group two-tailed Student´s t-test. For comparison, we show subunit Vma6 of the membrane-embedded domain of the V-ATPase, which is not significantly enriched. For the individual dots to be clear, the range chosen for the X axis in panel A excludes the dot representing the protein Cwp1, which showed a Log2 (wt / oxr1Δ) of -2,367955 and a Log10 (Intensity) of 6.705487389. For the individual dots to be clear, the range chosen for the X axis in panel B excludes the dots representing the proteins Rtc5 and Isw2. Rtc5: Log2 (wt / rtc5Δ) = 3.071144827 and a Log10 (Intensity) = 7.862733486. Isw2: Log2 (wt / oxr1Δ) = 3.884988578 and a Log10 (Intensity) = 7.25197871.

.

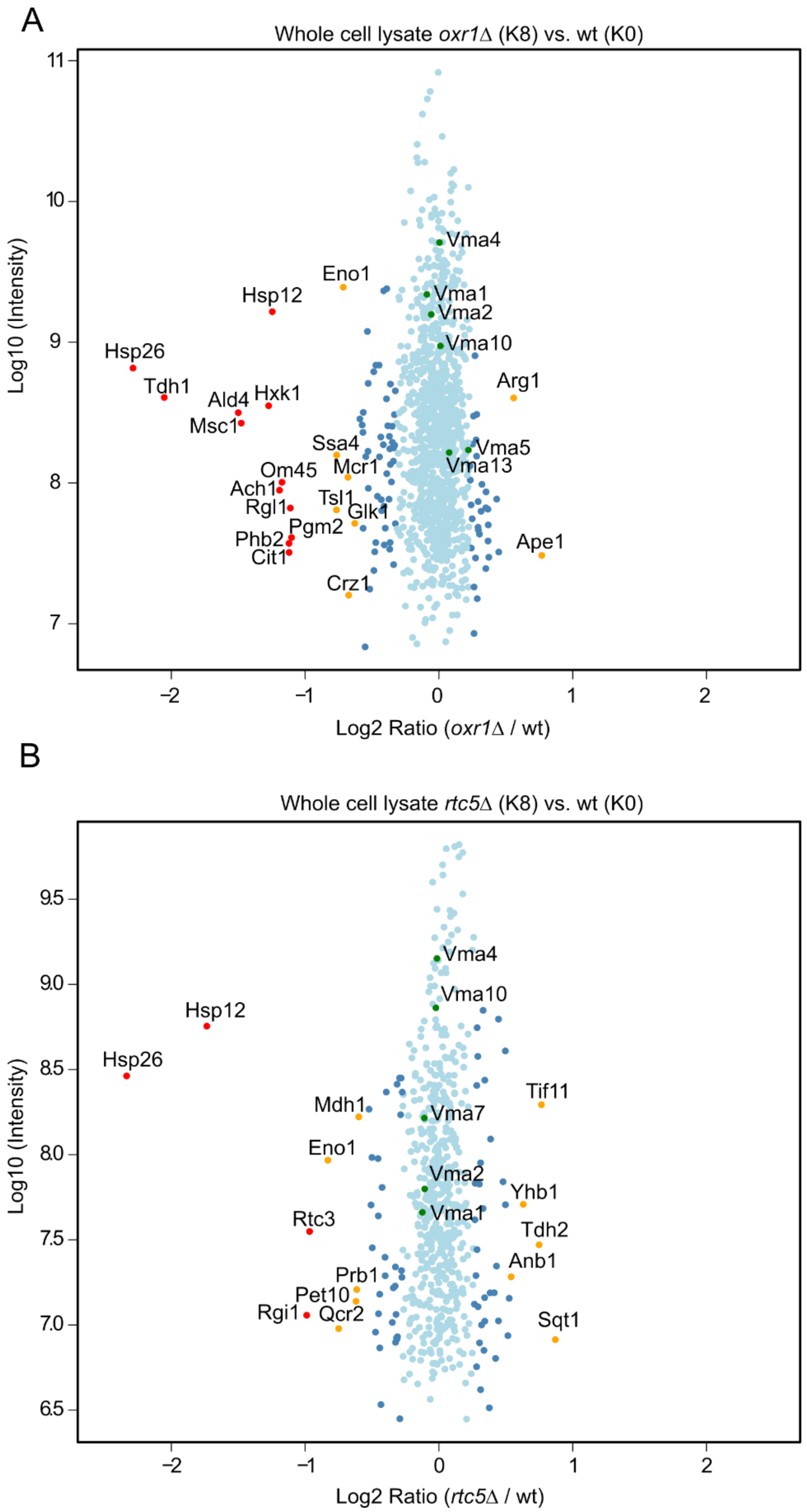

Appendix Figure S4: Whole cell proteomics of *oxr1*Δ and *rtc5*Δ strains
A and B) SILAC-based whole cell proteomics of cells lacking either *OXR1* (A) or *RTC5* (B) compared with the wt strain. Log10 of the detected protein intensities are plotted against Log2 of the heavy/light SILAC ratios. Significant outliers are color-coded in red ($P < 1e{-}14$), orange ($P < 0.0001$), or dark blue ($P < 0.05$); other identified proteins are shown in light blue. Statistical comparison is based on a two-group two-tailed Student´s t-test. V-ATPase subunits are labeled and shown as green dots.

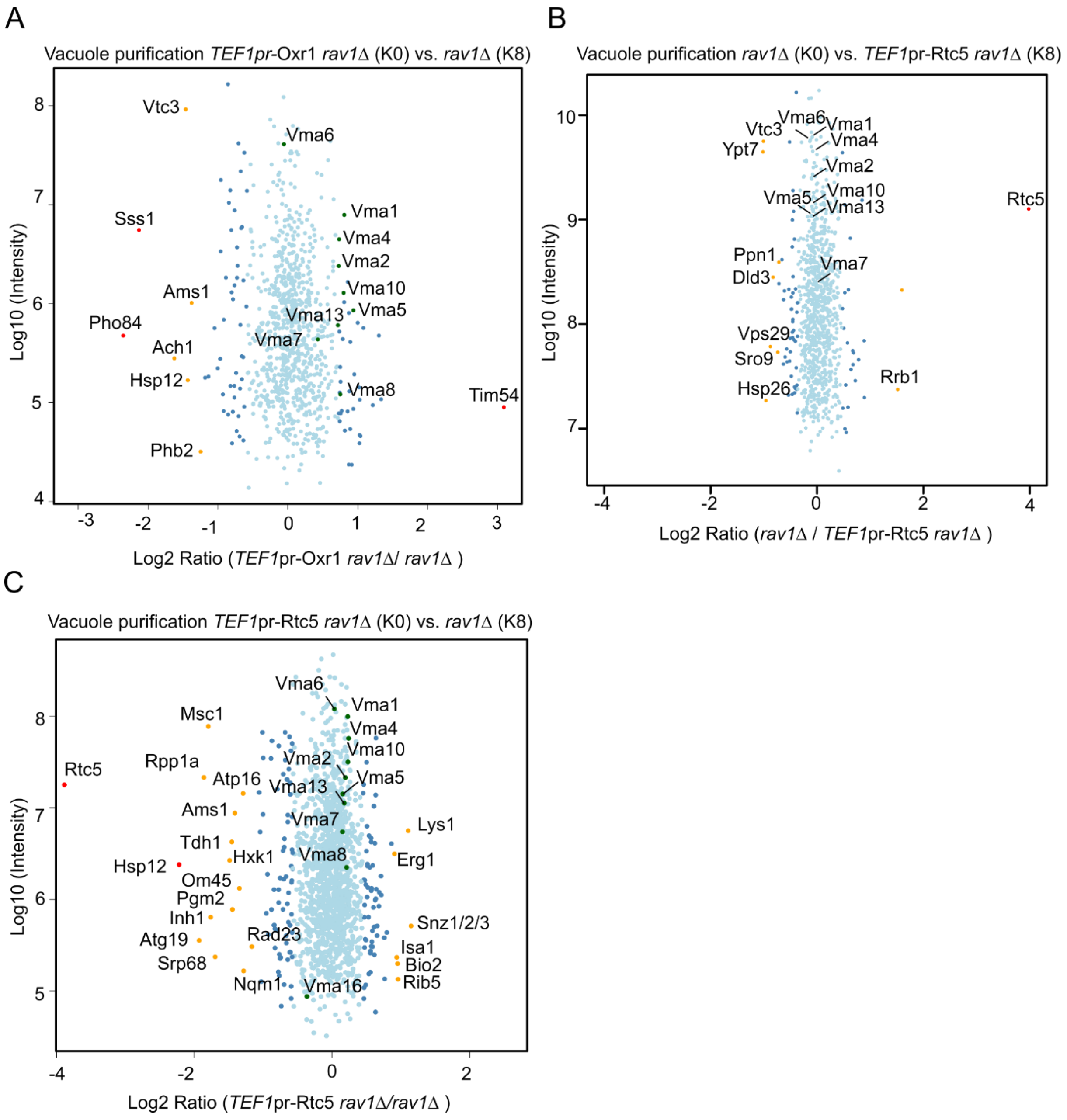

Appendix Figure S5: Vacuole proteomics of *rav1*Δ strains compared with *rav1*Δ strains overexpressing either Rtc5 or Oxr1

A - C) SILAC-based vacuole proteomics of *rav1*Δ compared to *rav1Δ* cells that overexpress either Oxr1 (A) or Rtc5 (B and C). Log10 of the detected protein intensities are plotted against Log2 of the heavy/light SILAC ratios. Dots are color-coded according to significance based on a two-group two-tailed Student´s t-test, red ($P < 1e{-}14$), orange ($P < 0.0001$), dark blue ($P < 0.05$), light blue ($P > 0.05$).

For the individual dots to be clear, the range chosen for the X axis in panel B excludes the dot representing the protein Yta12, which showed a Log2 (*TEF1pr-Rtc5 rav1Δ / rav1Δ*) of 6.637523306 and a Log10 (Intensity) of 7.485281232.

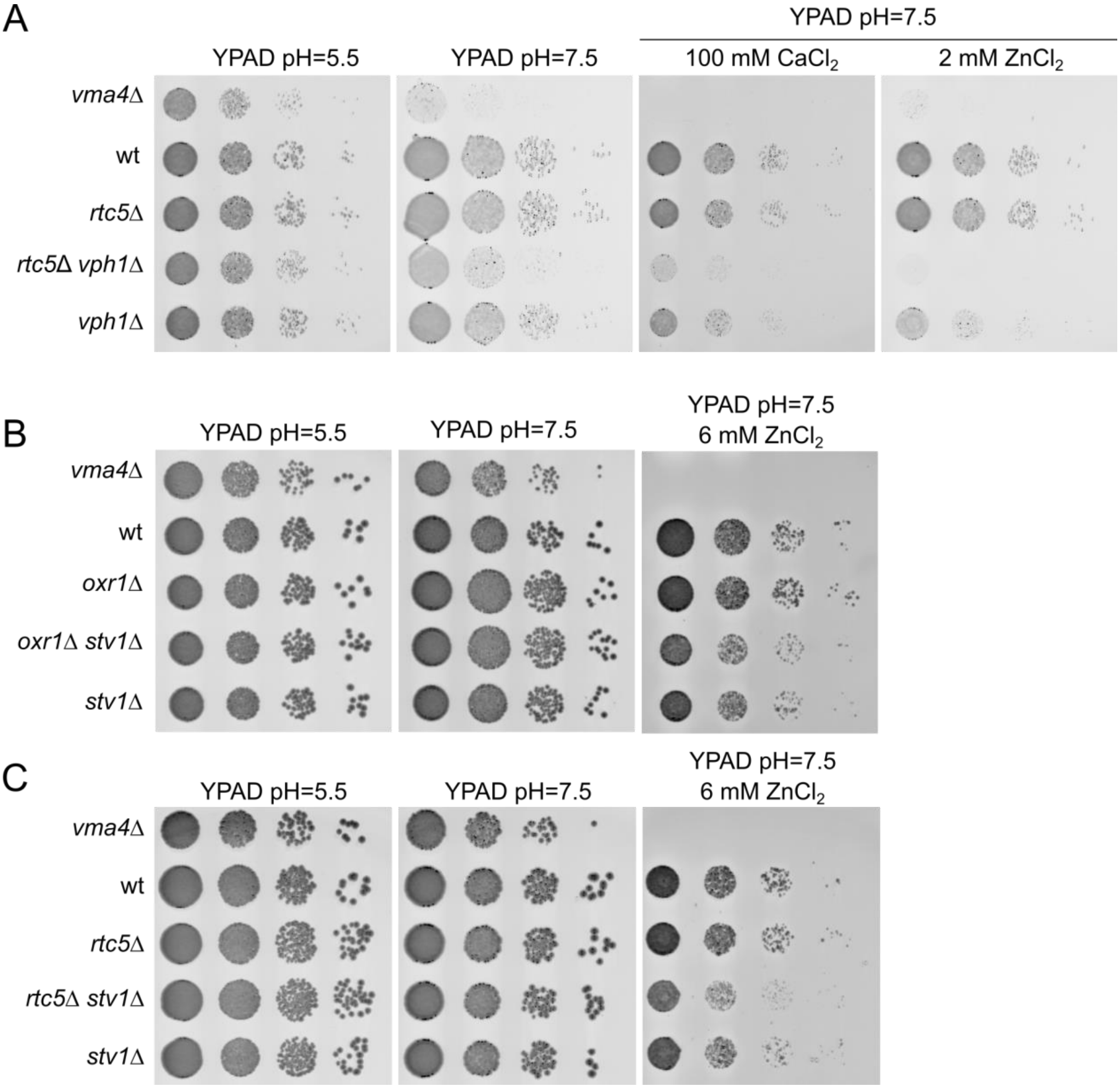

Appendix Figure S6: Genetic interactions between yeast TLDc domain-containing proteins and isoforms of V-ATPase subunit a

A) Strains of the indicated genotypes were spotted as serial dilutions on media with pH=5.5 or media with pH=7.5 with or without 2 mM $ZnCl_2$ or 100 mM $CaCl_2$.

B and C) Strains of the indicated genotypes were spotted as serial dilutions on media with pH=5.5 or media with pH=7.5 with or without 6 mM $ZnCl_2$.
