# Supplementary material for: Yeast TLDc domain proteins regulate assembly state and subcellular localization of the V-ATPase
Source: EMBO J. 2024 Apr 8;43(9):9. doi: 10.1038/s44318-024-00097-2 (PMC11066047; doi:10.1038/s44318-024-00097-2)
Supplement: Supplementary file 2 — Table EV1 [file 44318_2024_97_MOESM2_ESM.docx]

**Table EV 1: *Saccharomyces cerevisiae* strains used in this study**

| **Name** | **Identifier** | **Genotype** | **Source/ Reference** |
| --- | --- | --- | --- |
| BY4741 | AGMY002 | MATa his3∆1 leu2∆0 met15∆0 ura3∆0 | Euroscarf |
| BY4742 | AGMY003 | MATalpha his3Δ1 leu2Δ0 lys2Δ0 ura3Δ0 | Euroscarf |
| SEY6210 | AGMY001 | MATalpha leu2-3,112 ura3-52 his3-∆200 trp-∆901 lys2-801 suc2-∆9 GAL | Reggiori Lab |
| Vma2-msGFP2 | AGMY860 | BY4742- *VMA2*::msGFP2-NatN2 | This study |
| Vph1-GFP | AGMY1335 | SEY6210-*VPH1*::GFP-hphNT1 | This study |
| Rtc5-mNeonGreen | AGMY377 | BY4741- *RTC5*::mNeonGreen-NatN2 | This study |
| Vma4∆ Rtc5-mNeonGreen | AGMY721 | BY4741- *vma4*∆::KanMx *RTC5*::mNeonGreen-HIS3 | This study |
| Rtc5-mNeonGreen Vma5∆ | AGMY1059 | BY4741- *RTC5*::mNeonGreen-NatN2 *vma4*∆::HphNT1 | This study |
| Vph1∆ | AGMY1332 | BY4741- *vph1*∆::KanMx | This study |
| Vph1∆ Rtc5-mNeonGreen | AGMY376 | BY4741- *vph1*∆::KanMx *RTC5*::mNeonGreen-NatN2 | This study |
| Vma11∆ | AGMY1333 | BY4741- *vma11*∆::KanMx | This study |
| Vma11∆ Rtc5-mNeonGreen | AGMY374 | BY4741- *vma11*∆::KanMx *RTC5*::mNeonGreen-NatN2 | This study |
| Vma5-msGFP2 | AGMY989 | BY4741- *VMA5*::msGFP2-NatN2 | This study |
| Vma2-msGFP2 | AGMY944 | BY4741- *VMA2*::msGFP2-NatN2 | This study |
| Rtc5-mNeonGreen Rav1∆ | AGMY1786 | BY4741- *RTC5*::mNeonGreen-NatN2 *rav1*∆::HphNT1 | This study |
| Rtc5-msGFP2 | AGMY070 | BY4741- *RTC5*::msGFP2-HIS3 | This study |
| *TEF1*pr-Rtc5-msGFP2 | AGMY340 | BY4741- *RTC5*::msGFP2-HIS3 *RTC5pr*::KANMx-*TEF1pr* | This study |
| *TEF1*pr-msGFP2-Rtc5 | AGMY361 | BY4741- *RTC5pr*::NatN2-*TEF1*pr-msGFP2 | This study |
| Rtc5(G2A)-mNeonGreen | AGMY698 | BY4741- RTC*5(G2A)*::mNeonGreen-HIS3 | This study |
| Rtc5-msGFP2 | AGMY372 | BY4741- *RTC5*::msGFP2-NatN2 | This study |
| Rtc5(G2A)-msGFP2 | AGMY716 | BY4741- *RTC5(G2A)*::msGFP2-NatN2 | This study |
| Vma4∆ | AGMY695 | BY4741- *vma4*∆::KanMx | This study |
| Rtc5∆ | AGMY571 | BY4741- *rtc5*∆::HphNT1 | This study |
| Oxr1∆ | AGMY397 | BY4741- *oxr1*∆::KanMx | This study |
| Rtc5∆ Oxr1∆ | AGMY796 | BY4741- *rtc5*∆::HphNT1 *oxr1*∆::KanMx | This study |
| Rtc5(G2A) | AGMY638 | BY4741- Rtc5*(G2A)* | This study |
| Vma5-msGFP2 Oxr1∆ | AGMY1787 | BY4741- *VMA5*::msGFP2-NatN2 *oxr1*∆::HphNT1 | This study |
| Vma5-msGFP2 Rtc5∆ | AGMY1789 | BY4741- *VMA5*::msGFP2-NatN2 *rtc5*∆::HphNT1 | This study |
| *TEF1*pr-Oxr1 | AGMY942 | BY4741- *OXR1pr*::KanMx-*TEF1pr* | This study |
| *TEF1*pr-Rtc5 | AGMY089 | BY4741- *RTC5pr*::KanMX-*TEF1pr* | This study |
| *TEF1*pr-Oxr1 *TEF1*pr-Rtc5 | AGMY1777 | BY4741- *OXR1pr*::KanMx-*TEF1pr RTC5pr*::NatN2-*TEF1pr* | This study |
| Vma5-msGFP2 *TEF1*pr-Oxr1 | AGMY1734 | BY4741- *VMA5*::msGFP2-NatN2 *OXR1pr*::KanMx-*TEF1pr* | This study |
| Vma5-msGFP2 *TEF1*pr-Rtc5 | AGMY1788 | BY4741- *VMA5*::msGFP2-NatN2 *RTC5pr*::URA3-*TEF1pr* | This study |
| Lys2∆ | AGMY371 | BY4741- *lys2*∆::HphNT1 | This study |
| Oxr1∆ Lys2∆ | AGMY993 | BY4741- *oxr1*∆::KanMx *lys2*∆::NATN2 | This study |
| Rtc5∆ Lys2∆ | AGMY991 | BY4741- *rtc5*∆::HphNT1 *lys2*∆::NATN2 | This study |
| Rav1∆ | AGMY1053 | BY4741- *rav1*∆::HphNT1 | This study |
| TEF1pr-Oxr1 Rav1∆ | AGMY1055 | BY4741-*OXR1pr*::KanMx-*TEF1pr* *rav1*∆::NatN2 | This study |
| TEF1pr-Oxr1 Lys2∆ | AGMY969 | BY4741-*OXR1pr*::KanMx-*TEF1pr* *lys2*∆::NatN2 | This study |
| TEF1pr-Oxr1 Lys2∆ Rav1∆ | AGMY1343 | BY4741-*OXR1pr*::KanMx-*TEF1pr* *lys2*∆::NatN2 *rav1*∆::HphNT1 | This study |
| TEF1pr-Rtc5 Rav1∆ | AGMY1054 | BY4741-*RTC5pr*::KanMX-*TEF1pr* *rav1*∆::NatN2 | This study |
| TEF1pr-Rtc5 Lys2∆ | AGMY968 | BY4741-*RTC5pr*::KanMX-*TEF1pr* *lys2*∆::NatN2 | This study |
| TEF1pr-Rtc5 Lys2∆ Rav1∆ | AGMY1344 | BY4741-*RTC5pr*::KanMX-*TEF1pr* *lys2*∆::NatN2 *rav1*∆::HphNT1 | This study |
| Lys2∆ Rav1∆ | AGMY1342 | BY4741- *lys2*∆::HphNT1 *rav1*∆::KanMx | This study |
| TEF1pr-Rtc5-mNeonGreen Rav1∆ | AGMY1201 | BY4741-*RTC5pr*::KanMX-*TEF1pr-RTC5*-mNeonGreen-HIS3 *rav1*∆::NatN2 | This study |
| TEF1pr-Rtc5-msGFP2 Rav1∆ | AGMY1833 | BY4741-*RTC5pr*::KanMX-*TEF1pr-RTC5*-msGFP2-NatN2 *rav1*∆::HphNT1 |  |
| Rav1∆ Oxr1∆ | AGMY1341 | BY4741- *rav1*∆::HphNT1 *oxr1*∆::NatN2 | This study |
| Rav1∆ Rtc5∆ | AGMY1340 | BY4741- *rav1*∆::HphNT1 *rtc5*∆::NatN2 | This study |
| Stv1∆ | AGMY1128 | BY4741- *stv1*∆::KanMX | This study |
| TEF1pr-Oxr1 Stv1∆ | AGMY1275 | BY4741-*OXR1pr*::KanMx-*TEF1pr* *stv1*∆::NatN2 | This study |
| TEF1pr-Rtc5 Stv1∆ | AGMY1274 | BY4741-*RTC5pr*::KanMX-*TEF1pr* *stv1*∆::NatN2 | This study |
| TEF1pr-Oxr1-msGFP2 | AGMY943 | BY4741- *OXR1pr*::KanMx-*TEF1pr-OXR1*-msGFP2-NatN2 | This study |
| TEF1pr-Oxr1-msGFP2 Rav1∆ | AGMY1352 | BY4741- *OXR1pr*::KanMx-*TEF1pr-OXR1*-msGFP2-NatN2 *rav1*∆::HphNT1 | This study |
| Oxr1-2xmNeonGreen | AGMY996 | BY4741- *OXR1*::2xmNeonGreen-KanMX | This study |
| Oxr1-2xmNeonGreen Rav1∆ | AGMY1354 | BY4741- *OXR1*::2xmNeonGreen-KanMX *rav1*∆:HphNT1 | This study |
| Stv1-mNeonGreen | AGMY693 | BY4741- *STV1*::mNeonGreen-HIS3 | This study |
| Stv1-mNeonGreen Sec7-2xmKate | AGMY1359 | BY4741-*STV1*::mNeonGreen-HIS3 *SEC7*::2xmKate-URA3 | This study |
| Stv1-mNeonGreen Sec7-2xmKate Pfa3-Halo | AGMY1551 | BY4741-*STV1*::mNeonGreen-HIS3 *SEC7*::2xmKate-URA3 *PFA3*::Halo-MET15 | This study |
| Stv1-mNeonGreen Oxr1∆ | AGMY1088 | BY4741-*STV1*::mNeonGreen-HIS3 *oxr1*∆::HphNT1 | This study |
| Stv1-mNeonGreen Oxr1∆ Sec7-2xmKate | AGMY1360 | BY4741-*STV1*::mNeonGreen-HIS3 *oxr1*∆::HphNT1 *SEC7*::2xmKate-URA3 | This study |
| Stv1-mNeonGreen Oxr1∆ Sec7-2xmKate Pfa3-Halo | AGMY1552 | BY4741-*STV1*::mNeonGreen-HIS3 *oxr1*∆::HphNT1 *SEC7*::2xmKate-URA3 *PFA3*::Halo-MET15 | This study |
| Stv1-mNeonGreen Rtc5∆ | AGMY1087 | BY4741-*STV1*::mNeonGreen-HIS3 *rtc5*∆::HphNT1 | This study |
| Stv1-mNeonGreen Rtc5∆ Sec7-2xmKate | AGMY1361 | BY4741-*STV1*::mNeonGreen-HIS3 *rtc5*∆::HphNT1 *SEC7*::2xmKate-URA3 | This study |
| Stv1-mNeonGreen Rtc5∆ Sec7-2xmKate Pfa3-Halo | AGMY1553 | BY4741-*STV1*::mNeonGreen-HIS3 *rtc5*∆::HphNT1 *SEC7*::2xmKate-URA3 *PFA3*::Halo-MET15 | This study |
| Stv1(1-452)-mNeonGreen | AGMY1364 | BY4741- *Stv1(1-452)*::mNeonGreen-HIS3 | This study |
| Stv1(1-452)-mNeonGreen Pfa3-Halo | AGMY1554 | BY4741- *Stv1(1-452)*::mNeonGreen-HIS3 *PFA3*::Halo-MET15 | This study |
| Oxr1∆ Stv1(1-452)-mNeonGreen | AGMY1365 | BY4741-*oxr1*∆::KanMx *Stv1(1-452)*::mNeonGreen-HIS3 | This study |
| Oxr1∆ Stv1(1-452)-mNeonGreen Pfa3-Halo | AGMY1555 | BY4741-*oxr1*∆::KanMx *Stv1(1-452)*::mNeonGreen-HIS3 *PFA3*::Halo-MET15 | This study |
| Vph1∆ Oxr1∆ | AGMY1130 | BY4741- *vph1*∆::KanMx *oxr1*∆::HphNT1 | This study |
| Rtc5∆ Vph1∆ | AGMY1198 | BY4741- *rtc5*∆::HphNT1 *vph1*∆::NatN2 | This study |
| Vph1∆ Stv1∆ | AGMY1129 | BY4741- *vph1*∆::KanMx *stv1*∆::HphNT1 | This study |
| Stv1-mNeonGreen Vph1∆ | AGMY1231 | BY4741-*STV1*::mNeonGreen-HIS3 *vph1*∆::NatN2 | This study |
| Oxr1∆ Stv1∆ | AGMY1273 | BY4741- *oxr1*∆::KanMx *stv1*∆::NatN2 | This study |
| Rtc5∆ Stv1∆ | AGMY1272 | BY4741- *rtc5*∆::HphNT1 *stv1*∆::NatN2 | This study |
| Vps10-GFP | AGMY1356 | BY4741- *VPS10*::GFP-KanMx | This study |
| Vps26∆ | AGMY1358 | BY4741- *vps26*∆::KanMx | Balderhaar et al, 2010 (Euroscarf library) |
| Vps26∆ Vps10-GFP | AGMY1357 | BY4741- *vps26*∆::KanMx *VPS10*::GFP-HIS3 | Balderhaar et al, 2010 |
| Vps10-GFP Oxr1∆ | AGMY1348 | BY4741-*VPS10*::GFP-KanMx *oxr1*∆::HphNT1 | This study |
| Kex2-GFP | AGMY1397 | BY4741- Kex2::GFP-HIS3 | This study |
| Kex2-GFP Oxr1∆ | AGMY1399 | BY4741- Kex2::GFP-HIS3 *oxr1*∆::NatN2 | This study |
| Kex2-GFP Vps26∆ | AGMY1398 | BY4741- Kex2::GFP-HIS3 *vps26*∆::NatN2 | This study |
