# Supplementary material for: Yeast TLDc domain proteins regulate assembly state and subcellular localization of the V-ATPase
Source: EMBO J. 2024 Apr 8;43(9):9. doi: 10.1038/s44318-024-00097-2 (PMC11066047; doi:10.1038/s44318-024-00097-2)
Supplement: Supplementary file 3 — Table EV2 [file 44318_2024_97_MOESM3_ESM.docx]

**Table EV 2: Oligonucleotides and plasmids used in this study**

|  | |  | | **Oligonucleotides used in this study** | | |  |  |
| --- | --- | --- | --- | --- | --- | --- | --- | --- |
| **Identifier** | **Sequence** | | | | | | | |
| oAGM345 | AATATTTGTTGTAGTATTATgttttagagctagaaatagcaagttaaaataagg | | | | | | | |
| oAGM346 | ATAATACTACAACAAATATTgatcatttatctttcactgcggag | | | | | | | |
| oAGM347 | AAGCAACCGGACCAGGTAATATTTGTTGTAGTTATATGGCACAGTCTTCTTCAATCAGTTCCAGTAACGAAGAGGGTTC | | | | | | | |
| oAGM034 | CGCATCGACGGCTACGGG | | | | | | | |
| oAGM035 | AGTGACCTGTTGCTGTGC | | | | | | | |
| **Plasmids used in this study** | | | | | | | | |
| **Name** | **Identifier** | | **Description** | | **Yeast selection marker** | **Source** | | |
| pRCC-K | pCU5003 | | Plasmid to introduce mutations using CRISPR/Cas9 | | Kan(G418) | (Generoso et al., 2016) | | |
| pRCC-K Rtc5 G2A | pAGM164 | | Plasmid to mutate Glycine2 to Alanine in the Rtc5 gene using CRISPR/Cas9 | | Kan(G418) | This study | | |
