# Supplementary material for: Yeast TLDc domain proteins regulate assembly state and subcellular localization of the V-ATPase
Source: EMBO J. 2024 Apr 8;43(9):9. doi: 10.1038/s44318-024-00097-2 (PMC11066047; doi:10.1038/s44318-024-00097-2)
Supplement: Supplementary file 4 — Table EV3 [file 44318_2024_97_MOESM4_ESM.docx]

**Table EV 3: Antibodies used in this study for western blot analysis**

| **Reagent** | **Source** | **Identifier** | **Dilution** |
| --- | --- | --- | --- |
| Mouse monoclonal anti-GFP | Roche | Cat# 11814460001  RRID: AB_390913 | 1: 1000 |
| Mouse monoclonal anti-Pgk1 | Invitrogen | Cat# 459250;  RRID: AB_2535525 | 1: 10000 |
| Rabbit polyclonal anti-Vam3 | Ungermann Lab | N/A | 1: 1000 |
| Rabbit polyclonal anti-Vac8 | Ungermann Lab | N / A | 1 : 3000 |
| DyLight 800 Goat anti Mouse IgG | Invitrogen | Cat# SA5-35521;  RRID: AB_2556774 | 1: 20000 |
| DyLight 800 Goat anti Rabbit IgG | Thermo Scientific | Cat# SA5-35571;  RRID: AB_614947 | 1: 20000 |
