# Supplementary figures and images for: Yeast TLDc domain proteins regulate assembly state and subcellular localization of the V-ATPase
Source: EMBO J. 2024 Apr 8;43(9):9. doi: 10.1038/s44318-024-00097-2 (PMC11066047; doi:10.1038/s44318-024-00097-2)

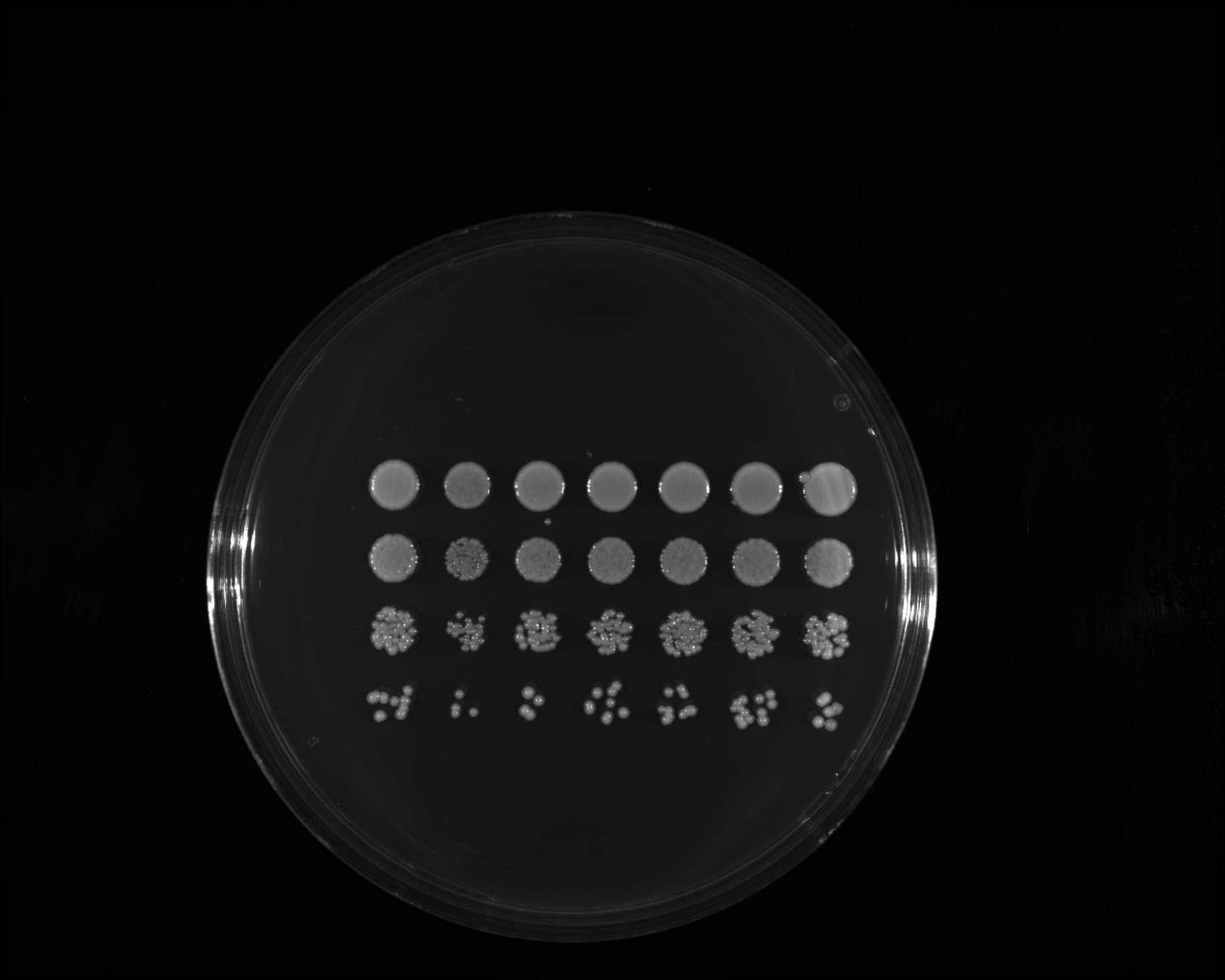

Supplement: Supplementary file 10 — Source data Fig. 5 [file 44318_2024_97_MOESM10_ESM.zip › Figure 5/5B/YPAD pH=5.5.tif]

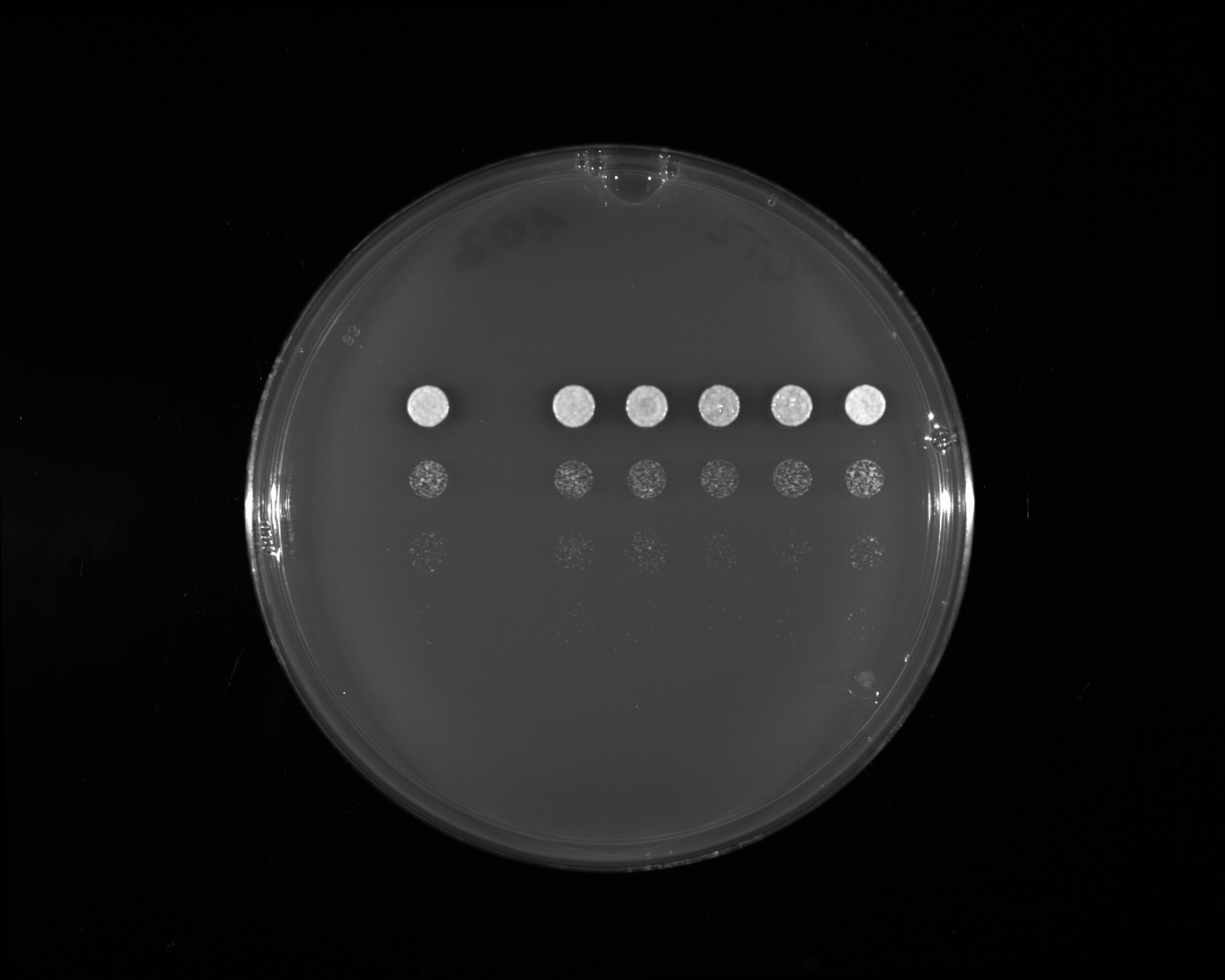

Supplement: Supplementary file 10 — Source data Fig. 5 [file 44318_2024_97_MOESM10_ESM.zip › Figure 5/5B/YPAD pH=7.5 10 mM ZnCl2.tif]

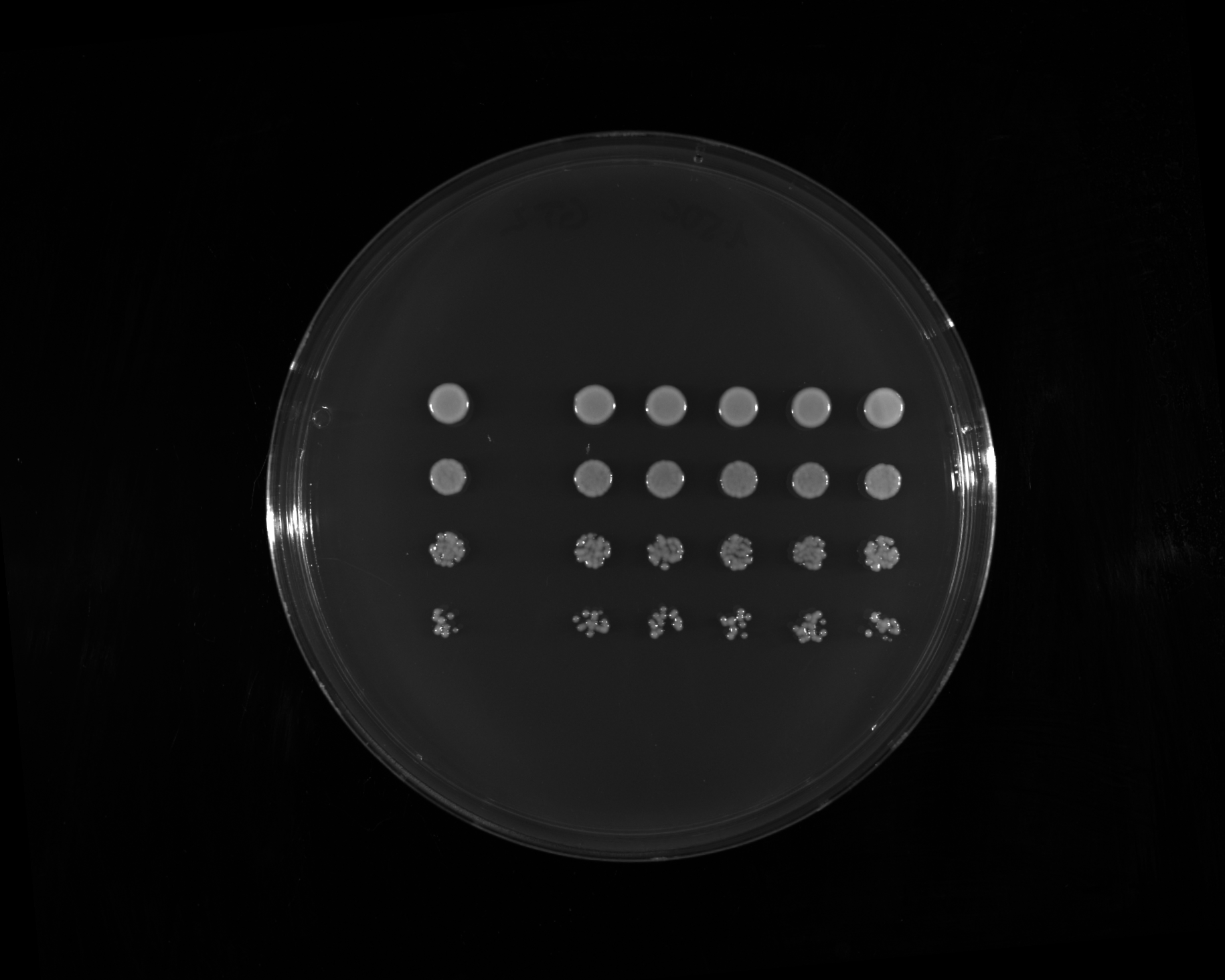

Supplement: Supplementary file 10 — Source data Fig. 5 [file 44318_2024_97_MOESM10_ESM.zip › Figure 5/5B/YPAD pH=7.5 150 mM CaCl2.tif]

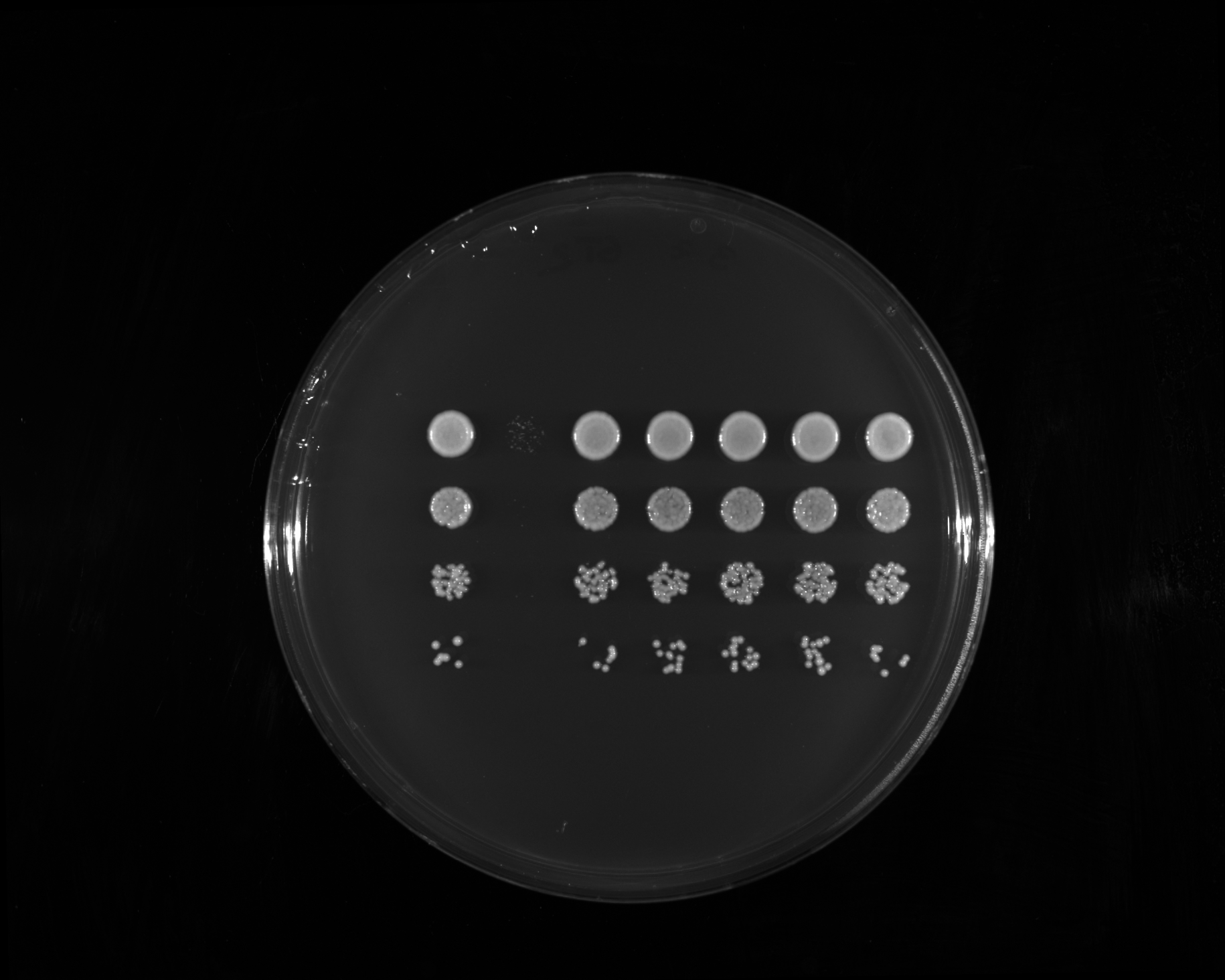

Supplement: Supplementary file 10 — Source data Fig. 5 [file 44318_2024_97_MOESM10_ESM.zip › Figure 5/5B/YPAD pH=7.5 3 mM ZnCl2.tif]

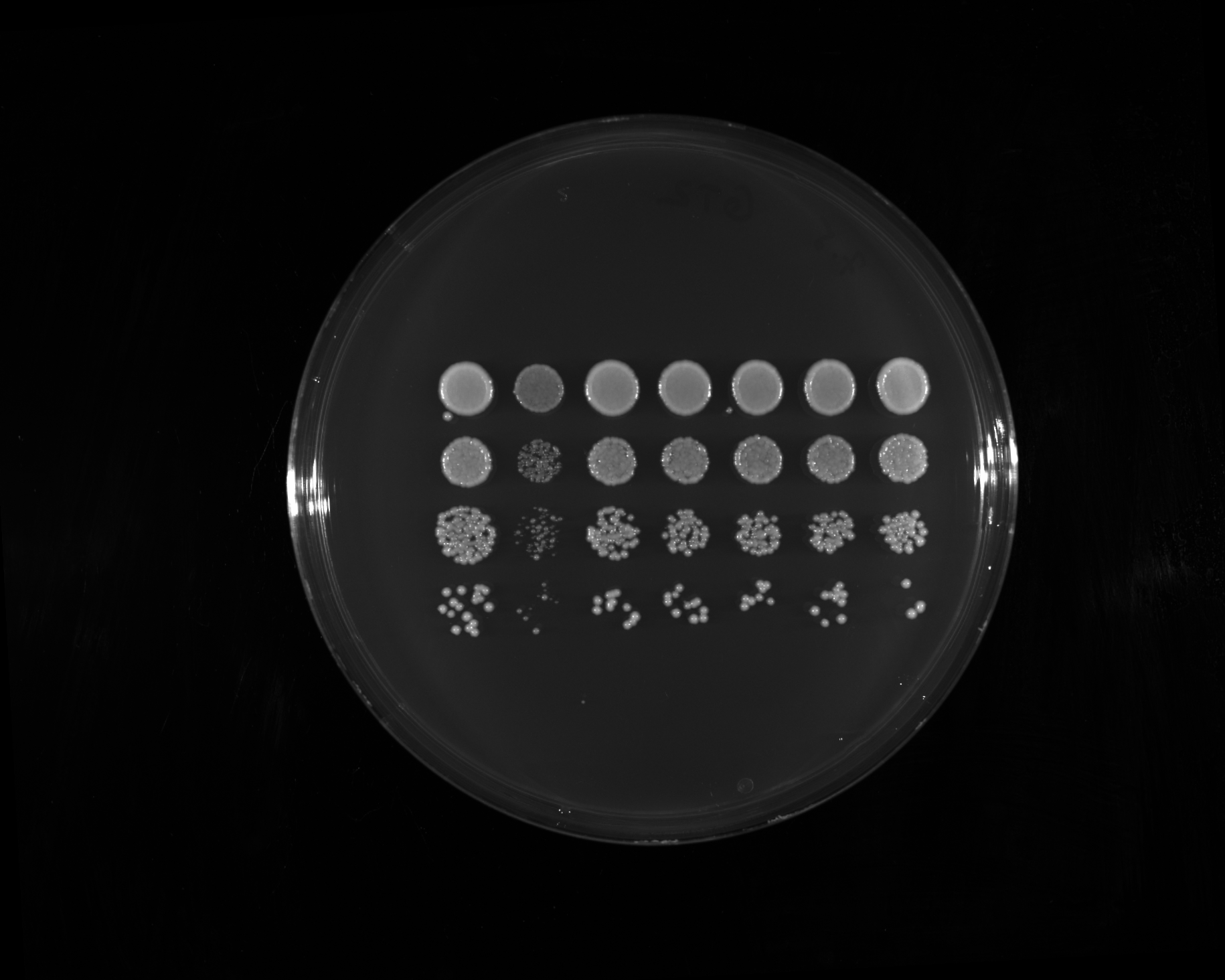

Supplement: Supplementary file 10 — Source data Fig. 5 [file 44318_2024_97_MOESM10_ESM.zip › Figure 5/5B/YPAD pH=7.5.tif]

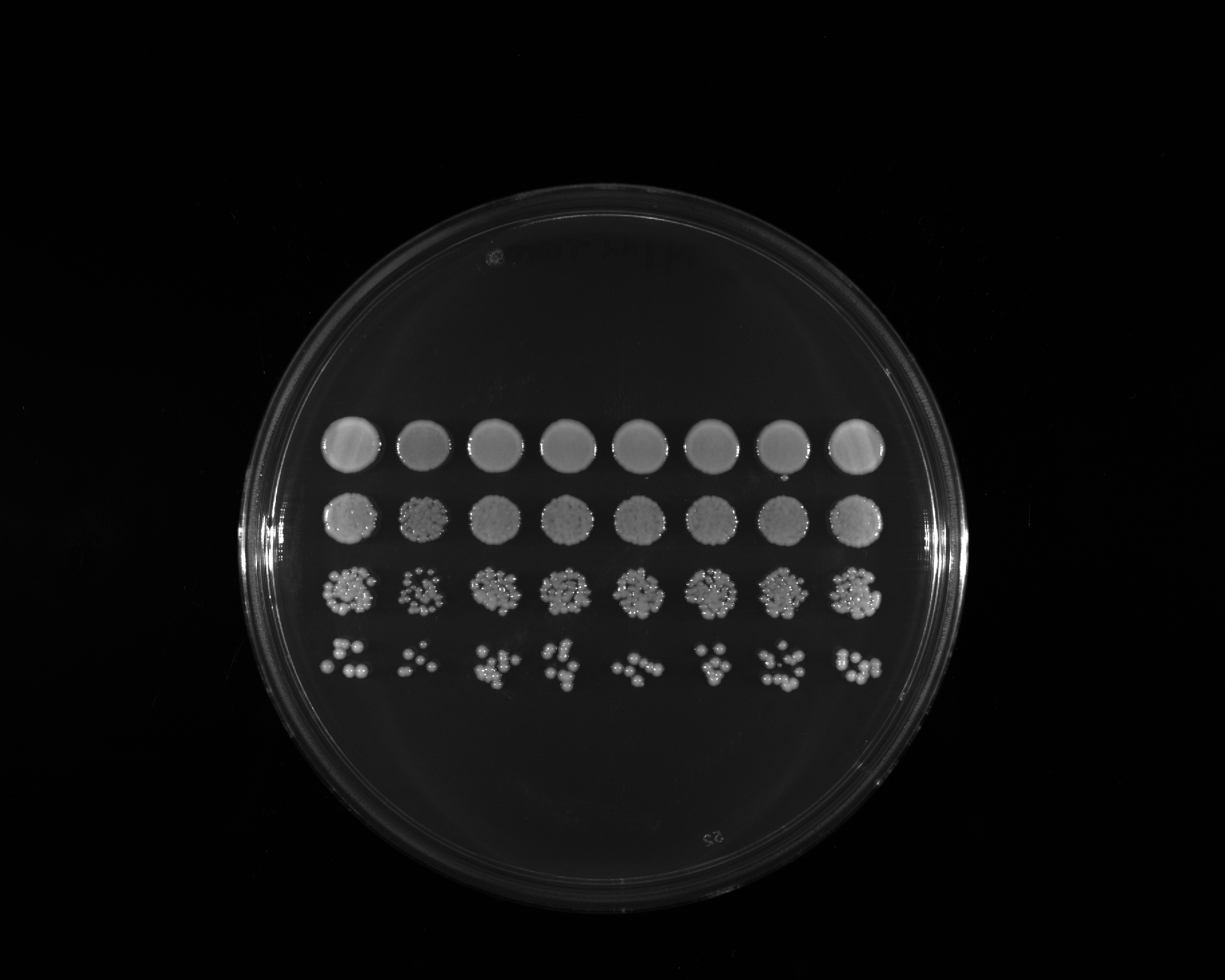

Supplement: Supplementary file 11 — Source data Fig. 6 [file 44318_2024_97_MOESM11_ESM.zip › Figure 6/6A/YPAD pH=5.5.tif]

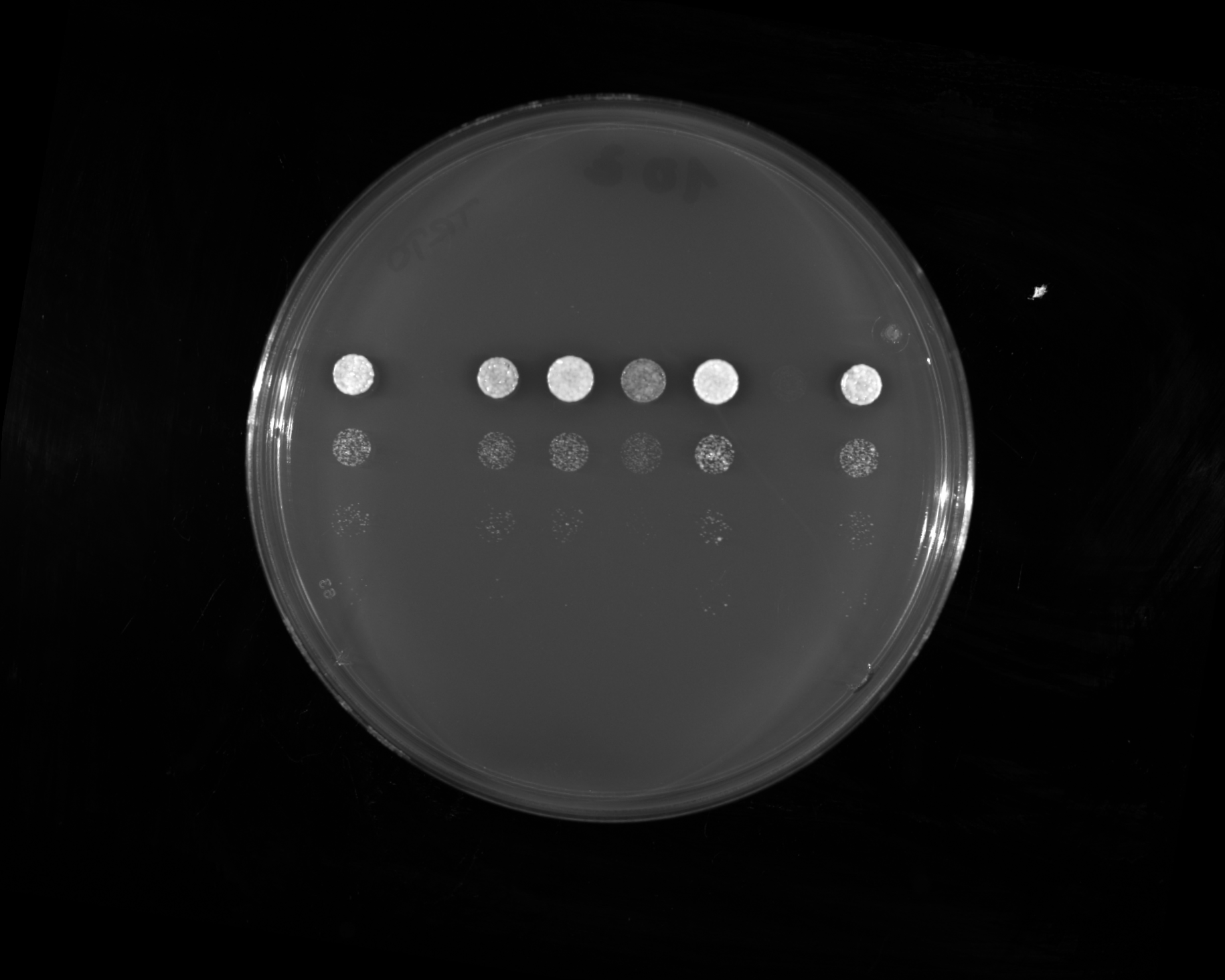

Supplement: Supplementary file 11 — Source data Fig. 6 [file 44318_2024_97_MOESM11_ESM.zip › Figure 6/6A/YPAD pH=7.5 10 mM ZnCl2.tif]

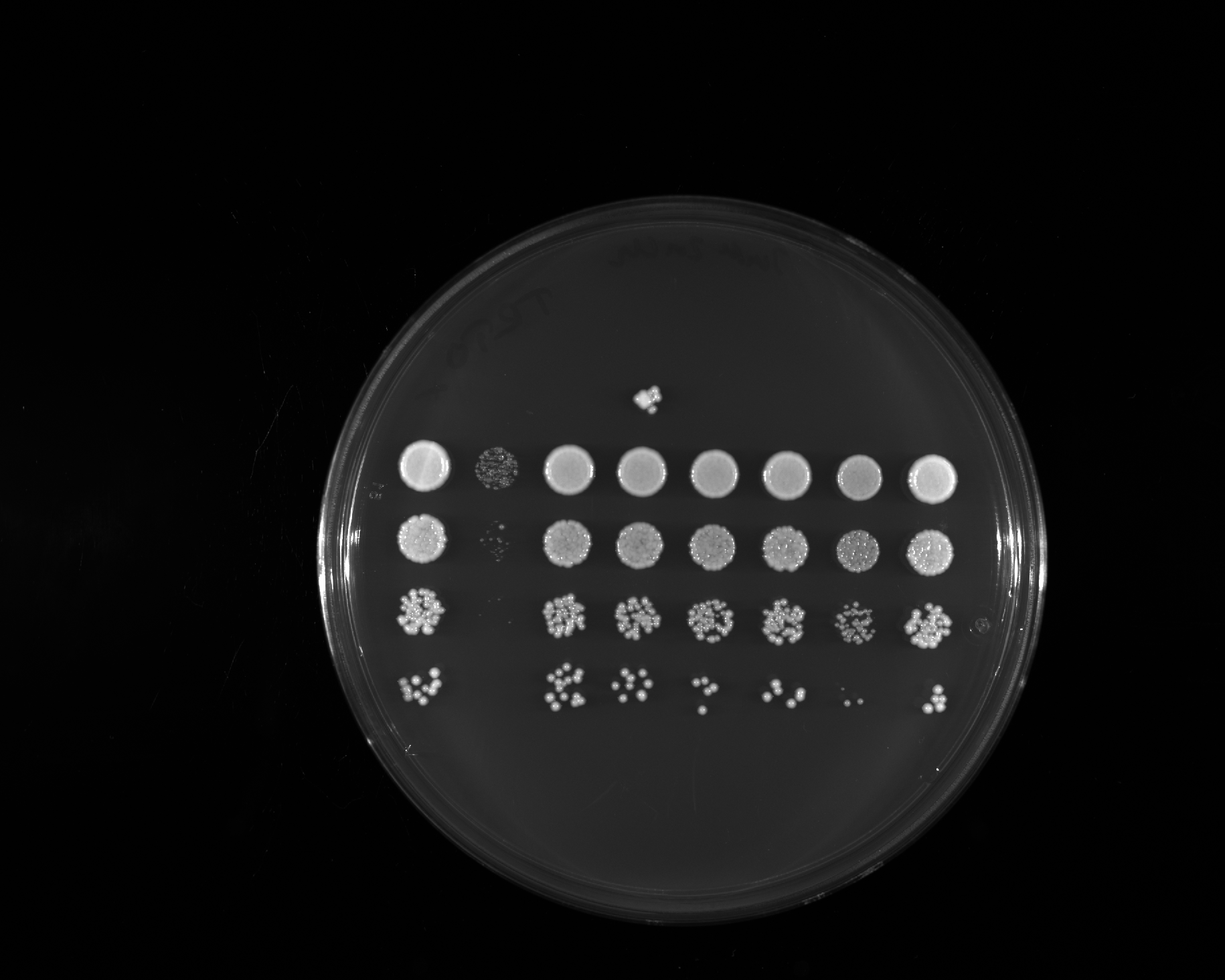

Supplement: Supplementary file 11 — Source data Fig. 6 [file 44318_2024_97_MOESM11_ESM.zip › Figure 6/6A/YPAD pH=7.5 3 mM ZnCl2.tif]

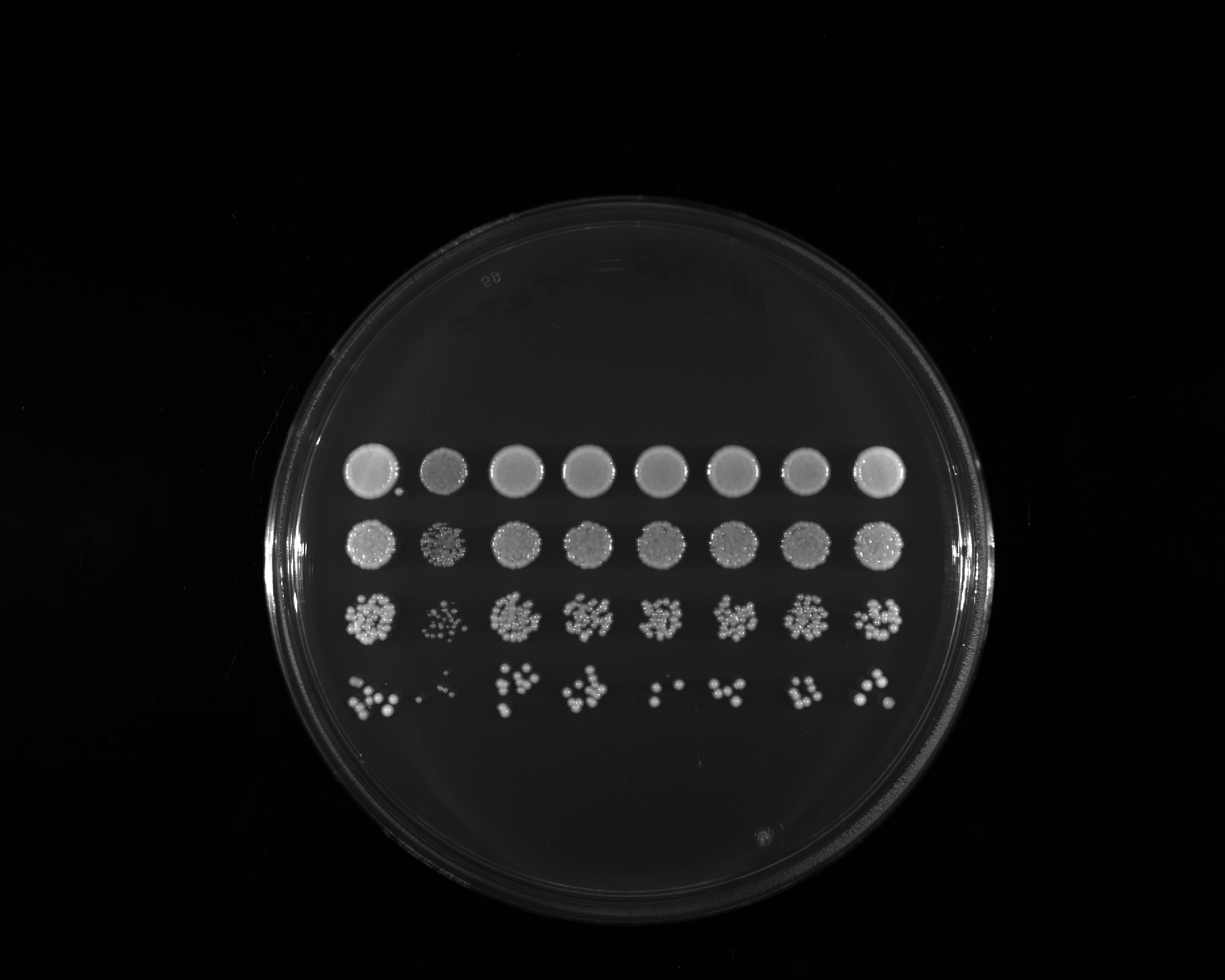

Supplement: Supplementary file 11 — Source data Fig. 6 [file 44318_2024_97_MOESM11_ESM.zip › Figure 6/6A/YPAD pH=7.5.tif]

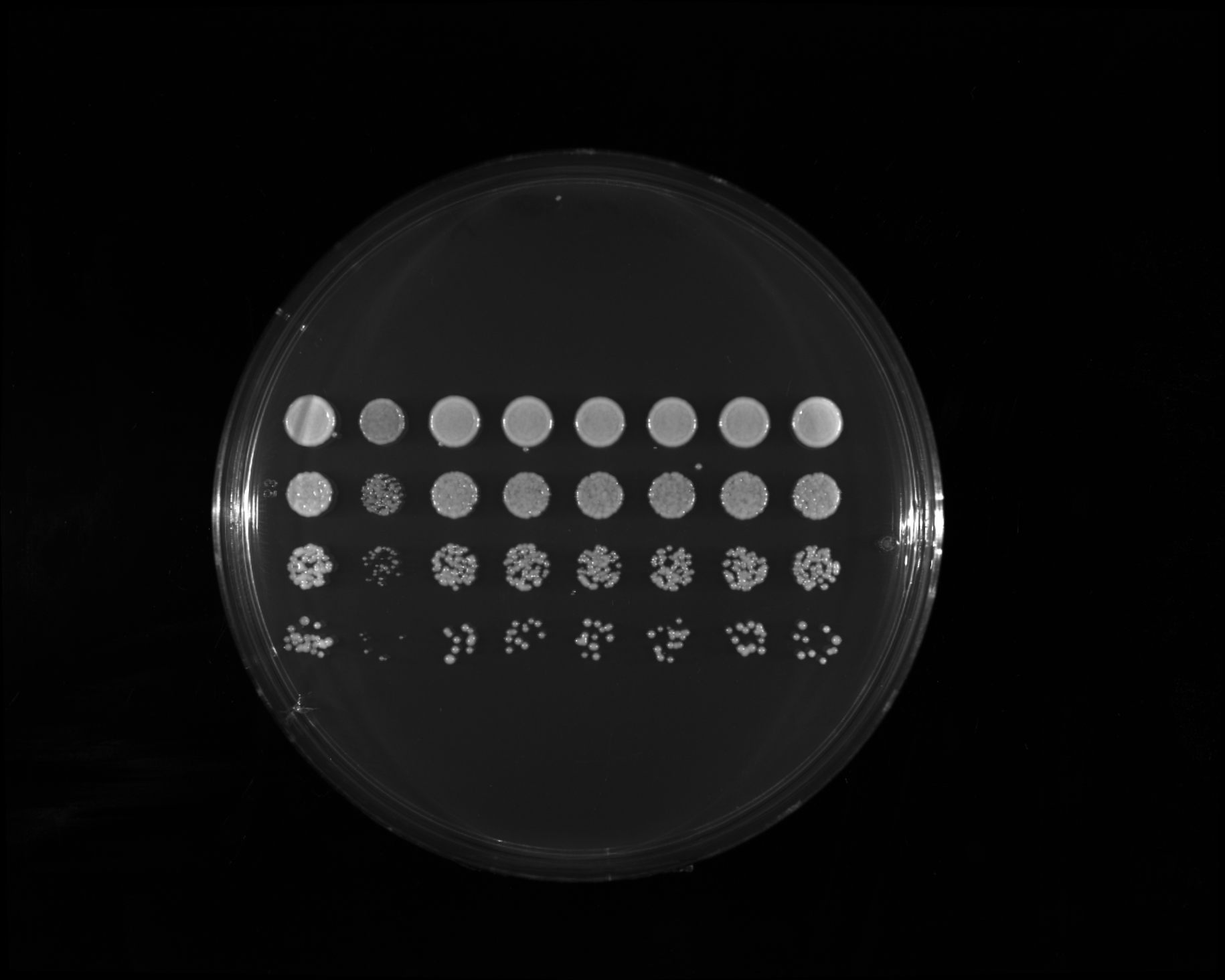

Supplement: Supplementary file 11 — Source data Fig. 6 [file 44318_2024_97_MOESM11_ESM.zip › Figure 6/6C/YPAG pH=5.5.tif]

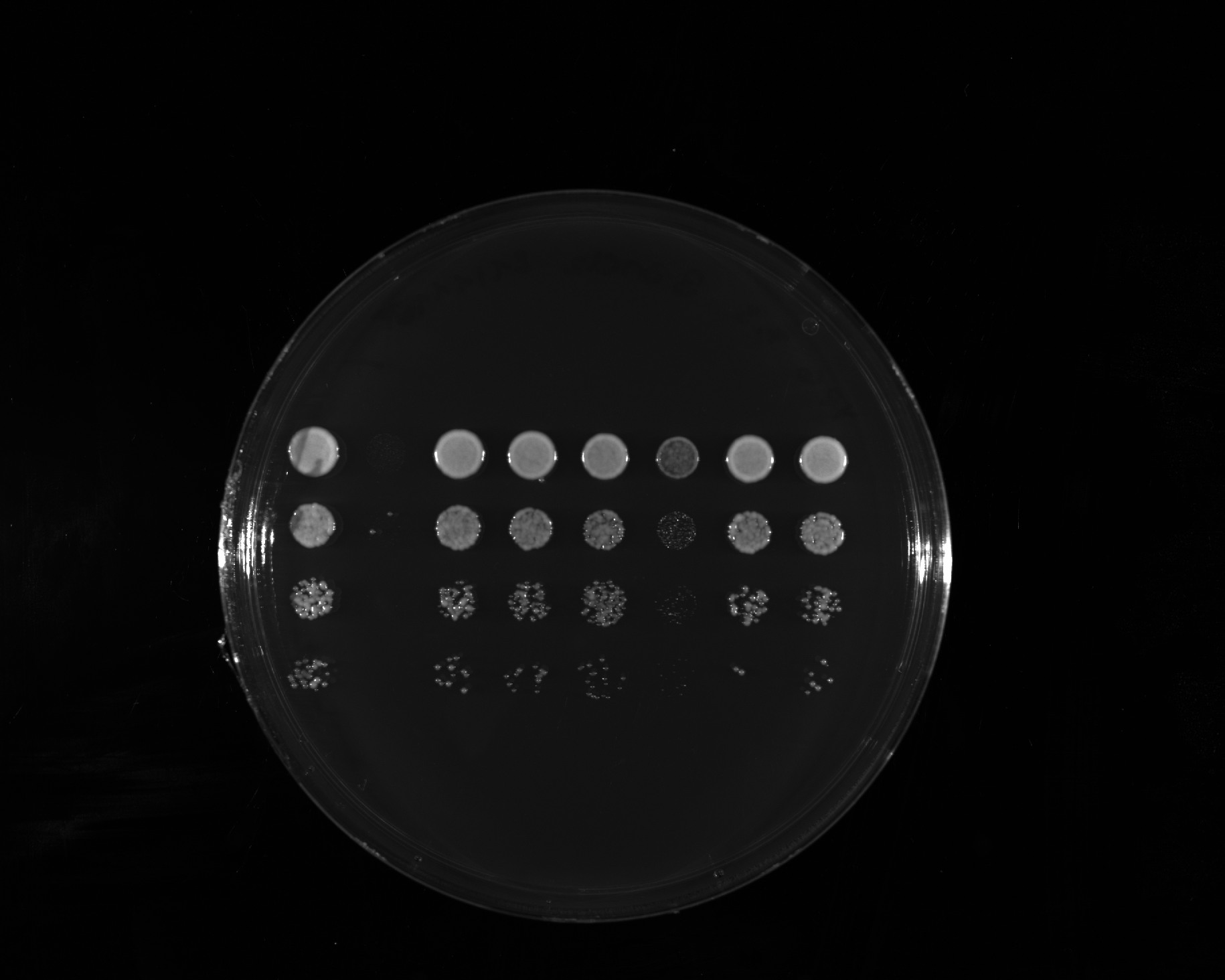

Supplement: Supplementary file 11 — Source data Fig. 6 [file 44318_2024_97_MOESM11_ESM.zip › Figure 6/6C/YPAG pH=7.5 3 mM ZnCl2.tif]

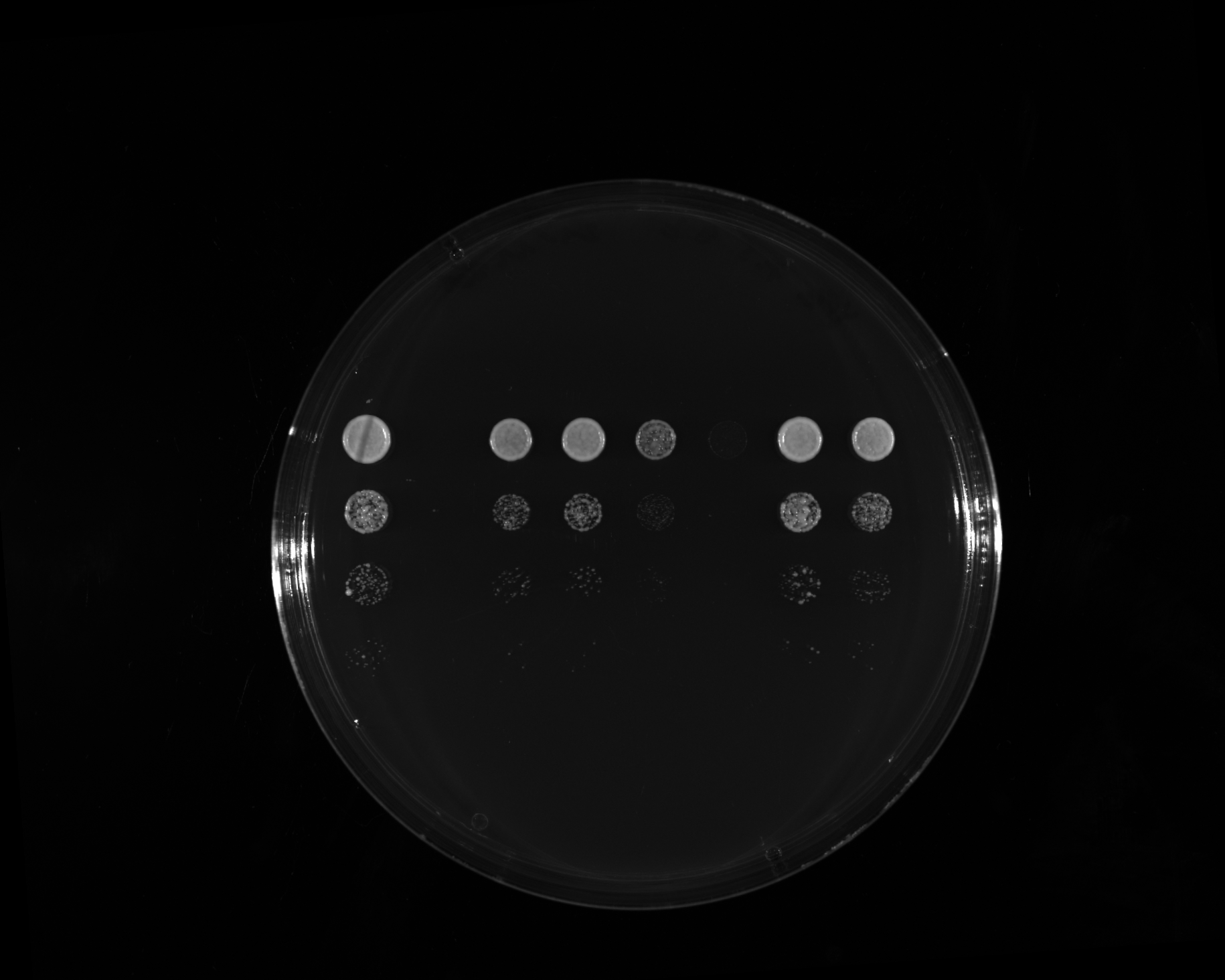

Supplement: Supplementary file 11 — Source data Fig. 6 [file 44318_2024_97_MOESM11_ESM.zip › Figure 6/6C/YPAG pH=7.5 8 mM ZnCl2.tif]

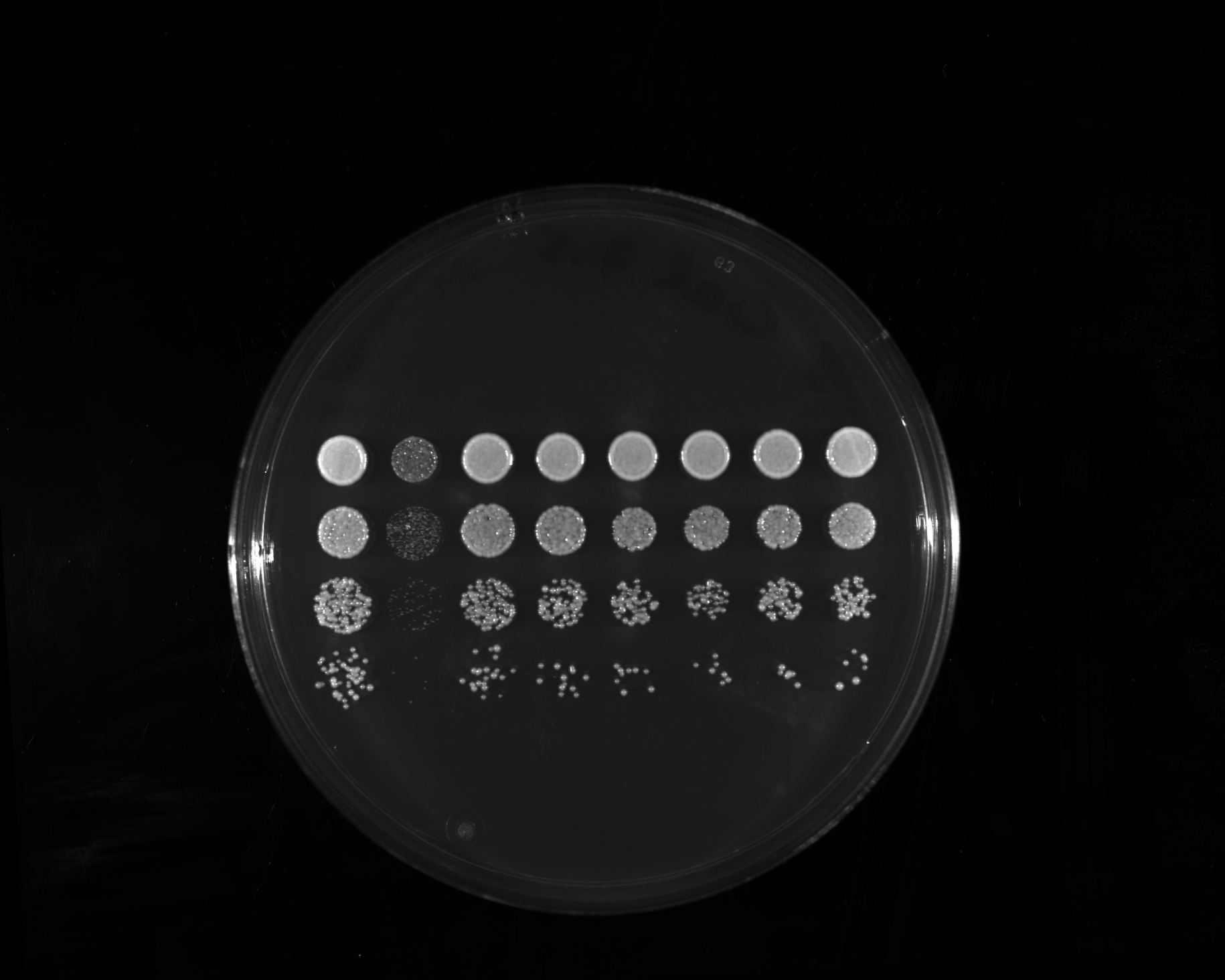

Supplement: Supplementary file 11 — Source data Fig. 6 [file 44318_2024_97_MOESM11_ESM.zip › Figure 6/6C/YPAG pH=7.5.tif]

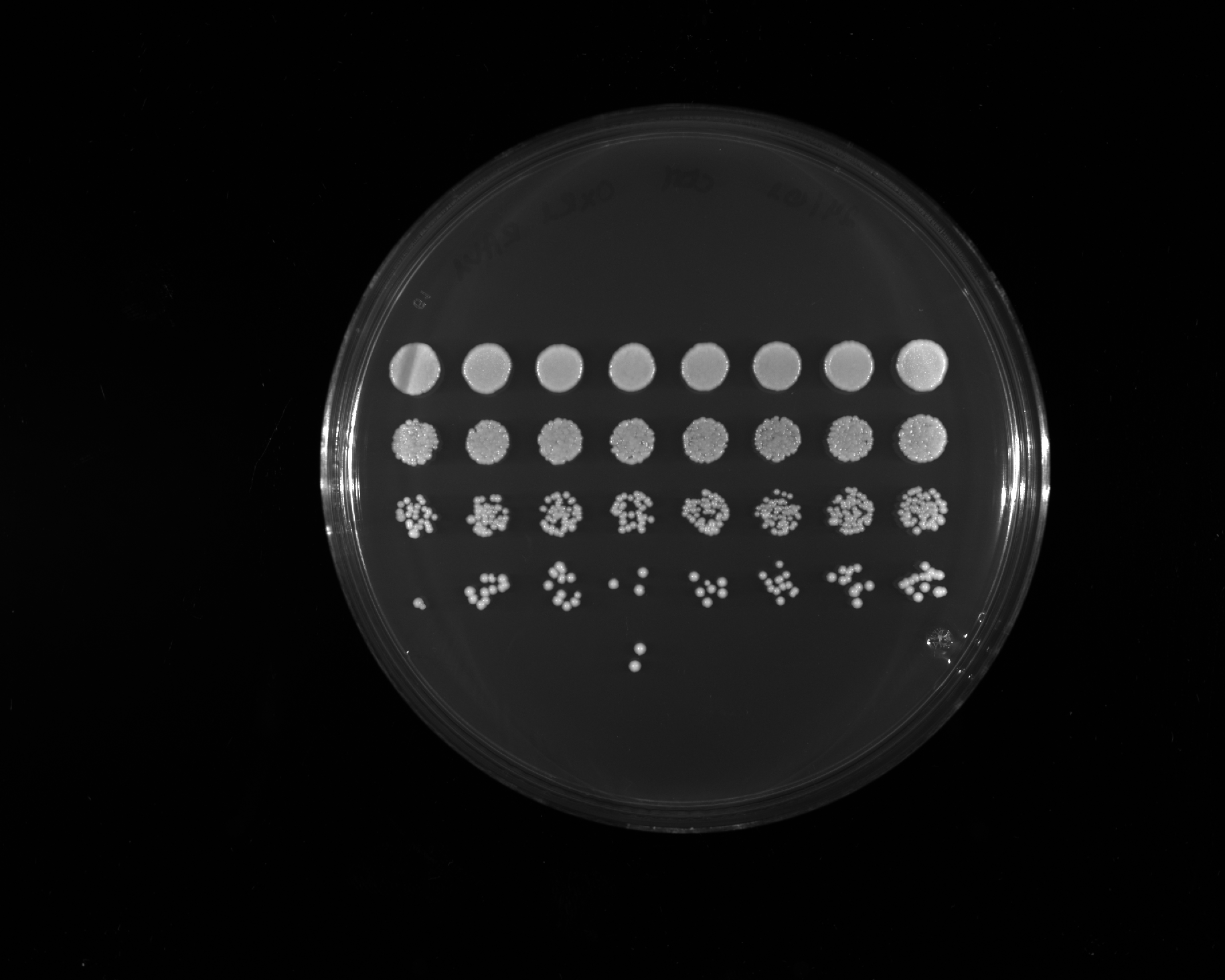

Supplement: Supplementary file 12 — Source data Fig. 7 [file 44318_2024_97_MOESM12_ESM.zip › Figure 7/7A/YPAD pH=5.5.tif]

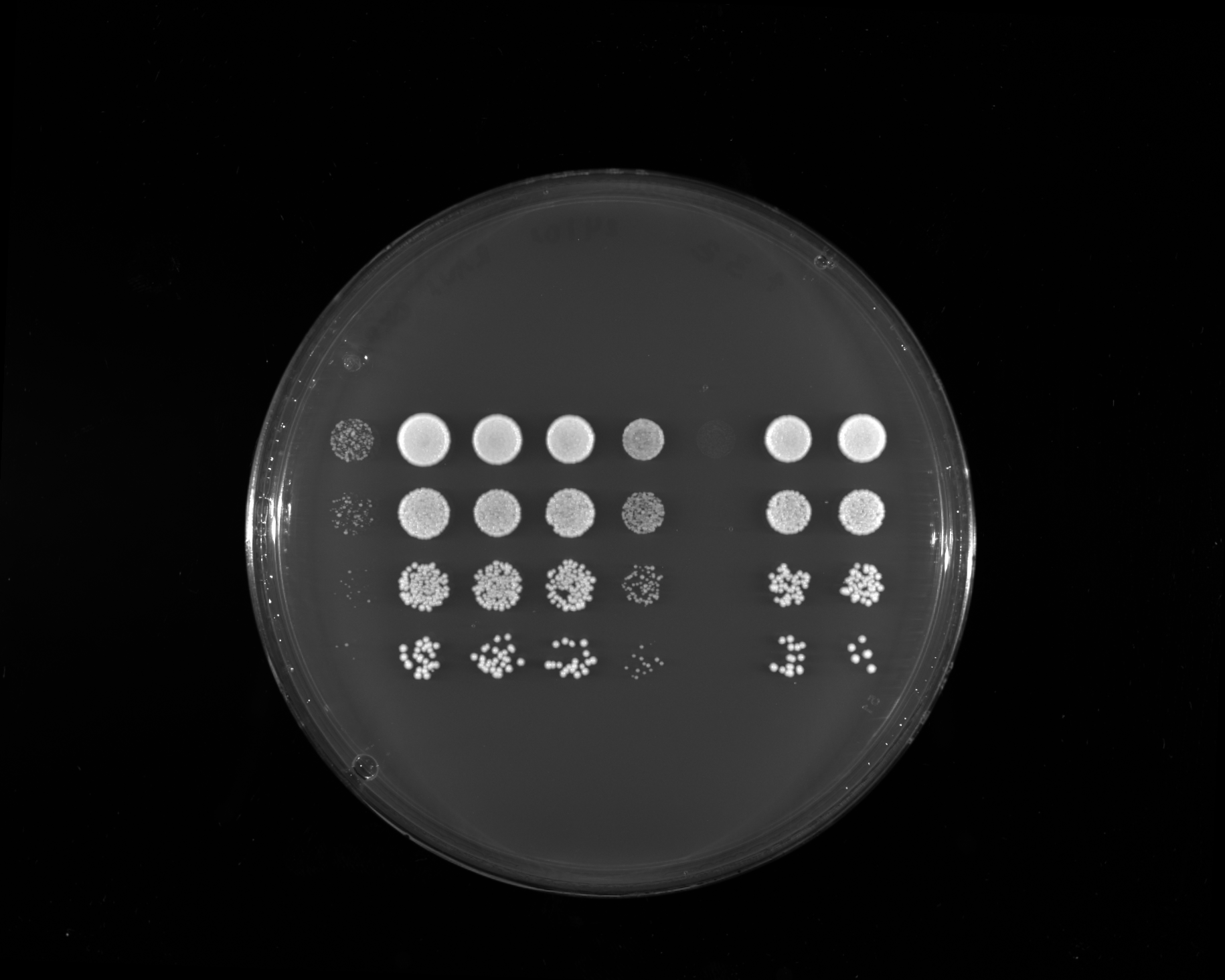

Supplement: Supplementary file 12 — Source data Fig. 7 [file 44318_2024_97_MOESM12_ESM.zip › Figure 7/7A/YPAD pH=7.5 3 mM ZnCl2.tif]

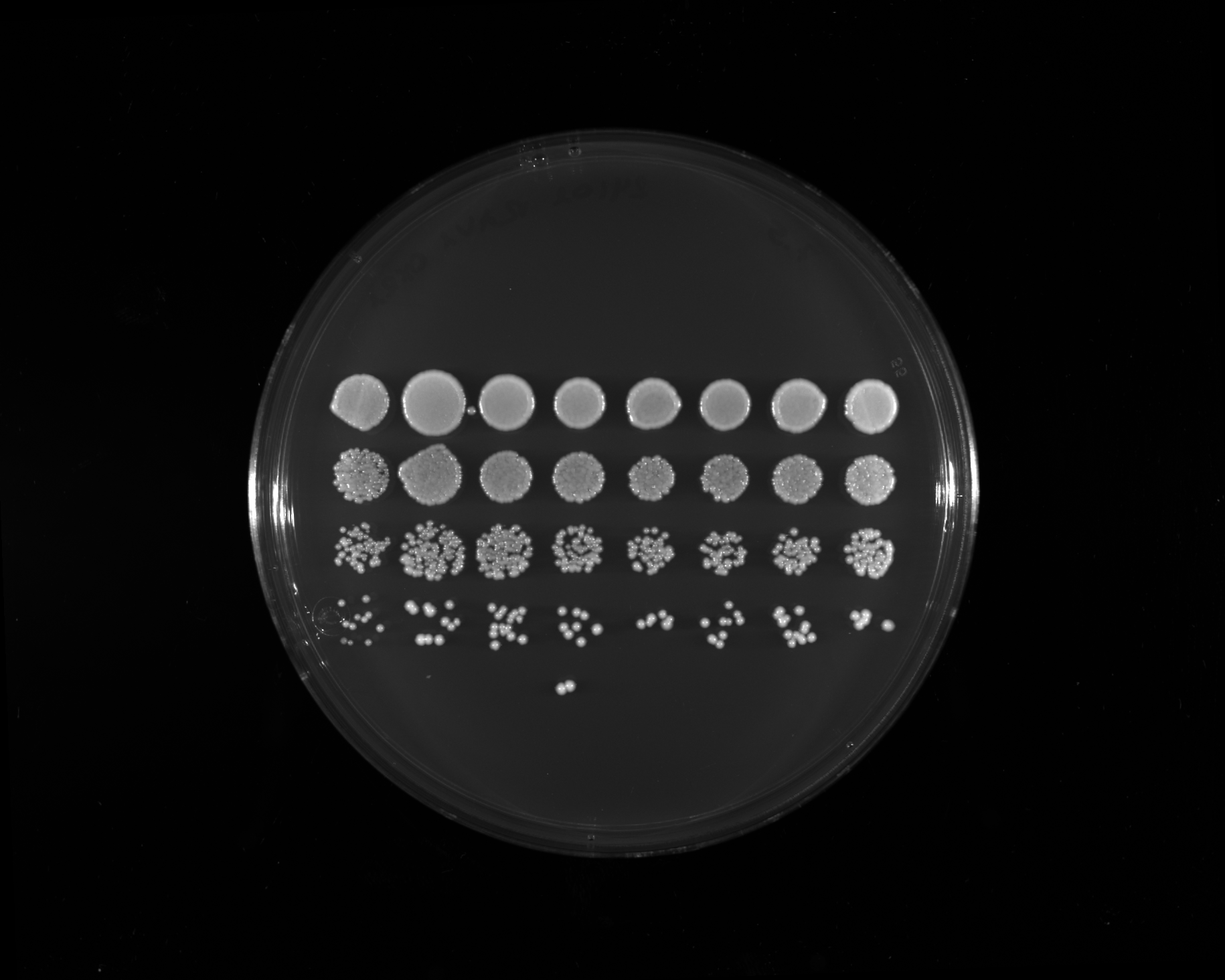

Supplement: Supplementary file 12 — Source data Fig. 7 [file 44318_2024_97_MOESM12_ESM.zip › Figure 7/7A/YPAD pH=7.5.tif]

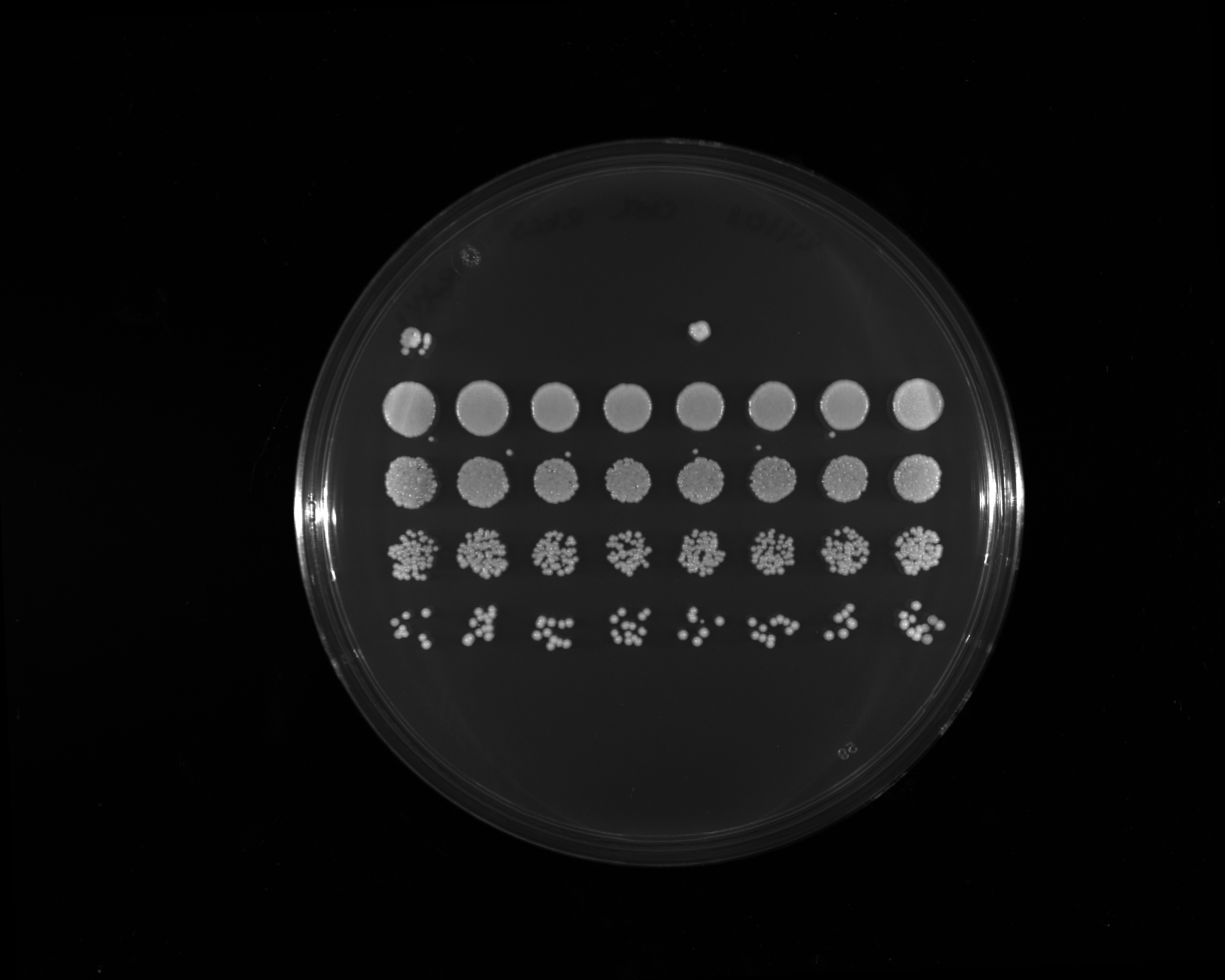

Supplement: Supplementary file 12 — Source data Fig. 7 [file 44318_2024_97_MOESM12_ESM.zip › Figure 7/7B/YPAD pH=5.5.tif]

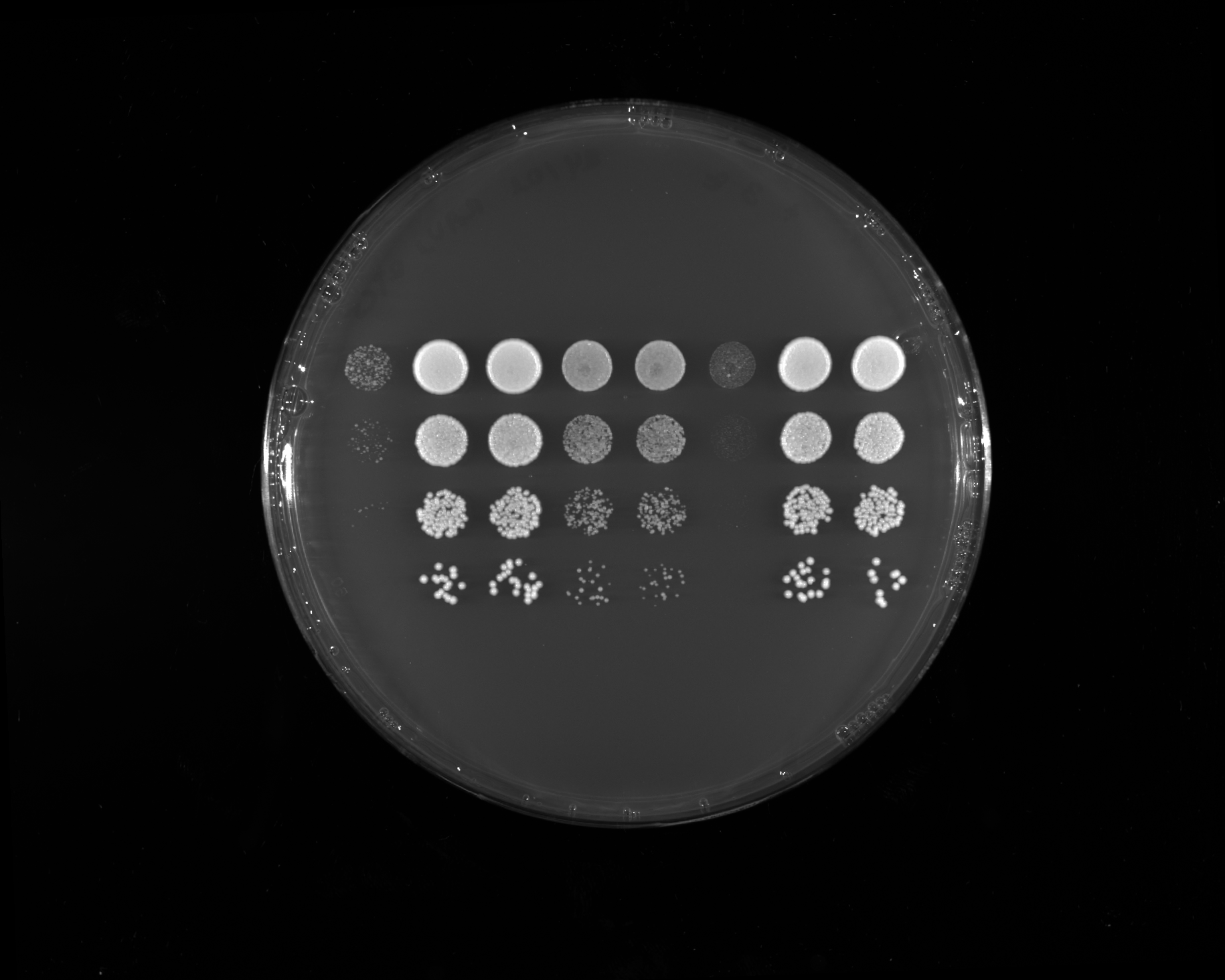

Supplement: Supplementary file 12 — Source data Fig. 7 [file 44318_2024_97_MOESM12_ESM.zip › Figure 7/7B/YPAD pH=7.5 3 mM ZnCl2.tif]

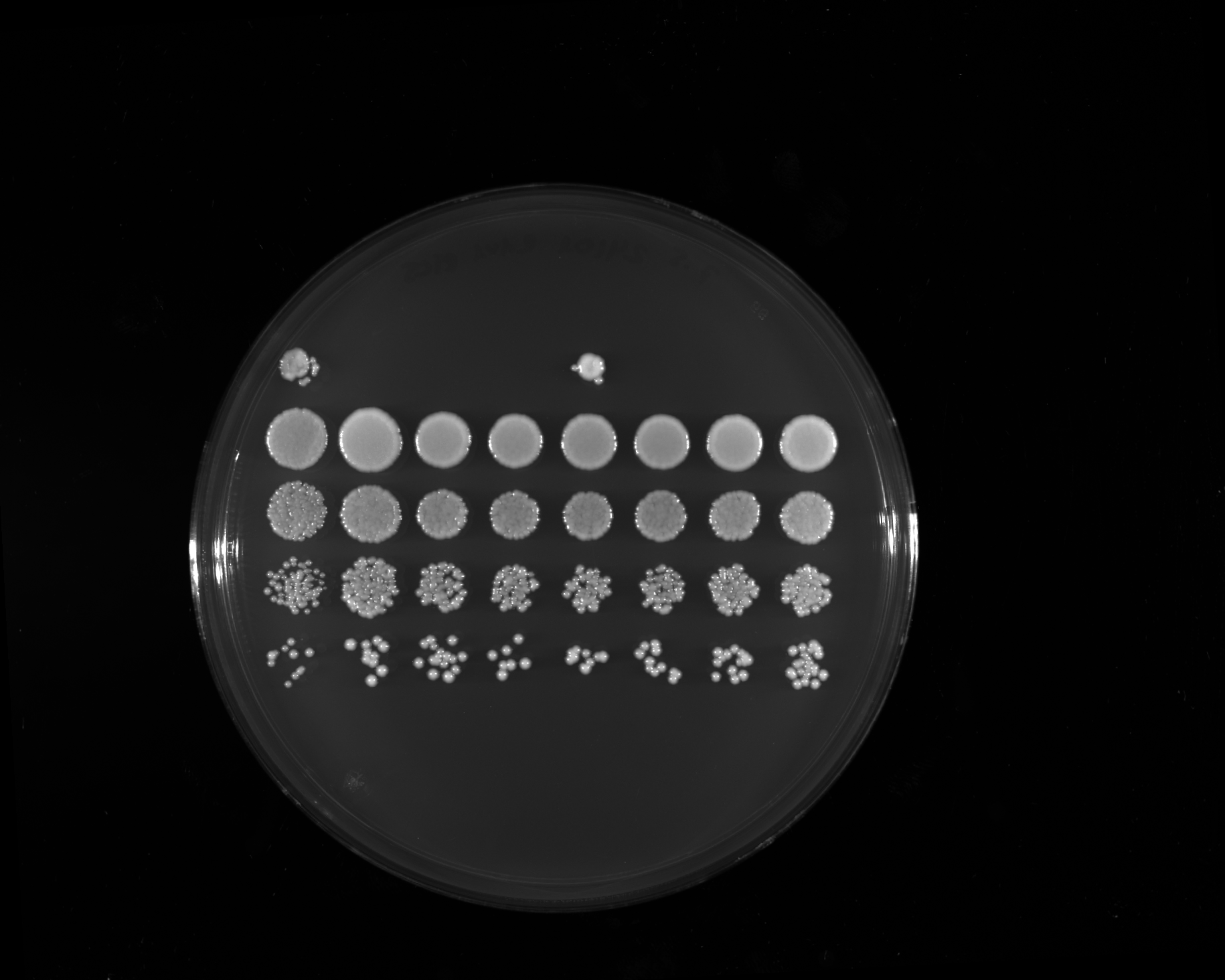

Supplement: Supplementary file 12 — Source data Fig. 7 [file 44318_2024_97_MOESM12_ESM.zip › Figure 7/7B/YPAD pH=7.5.tif]

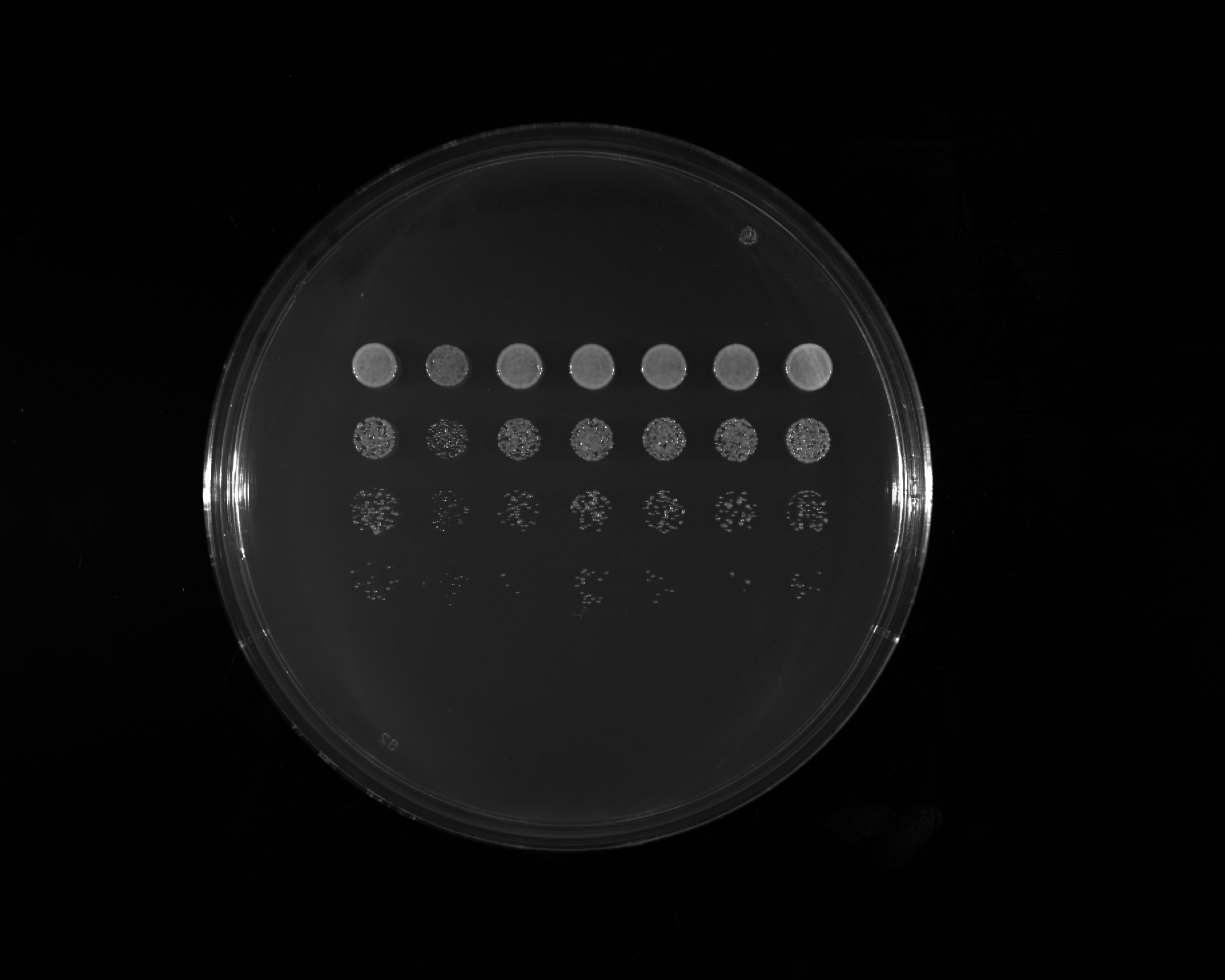

Supplement: Supplementary file 12 — Source data Fig. 7 [file 44318_2024_97_MOESM12_ESM.zip › Figure 7/7F/YPAD pH=5.5.tif]

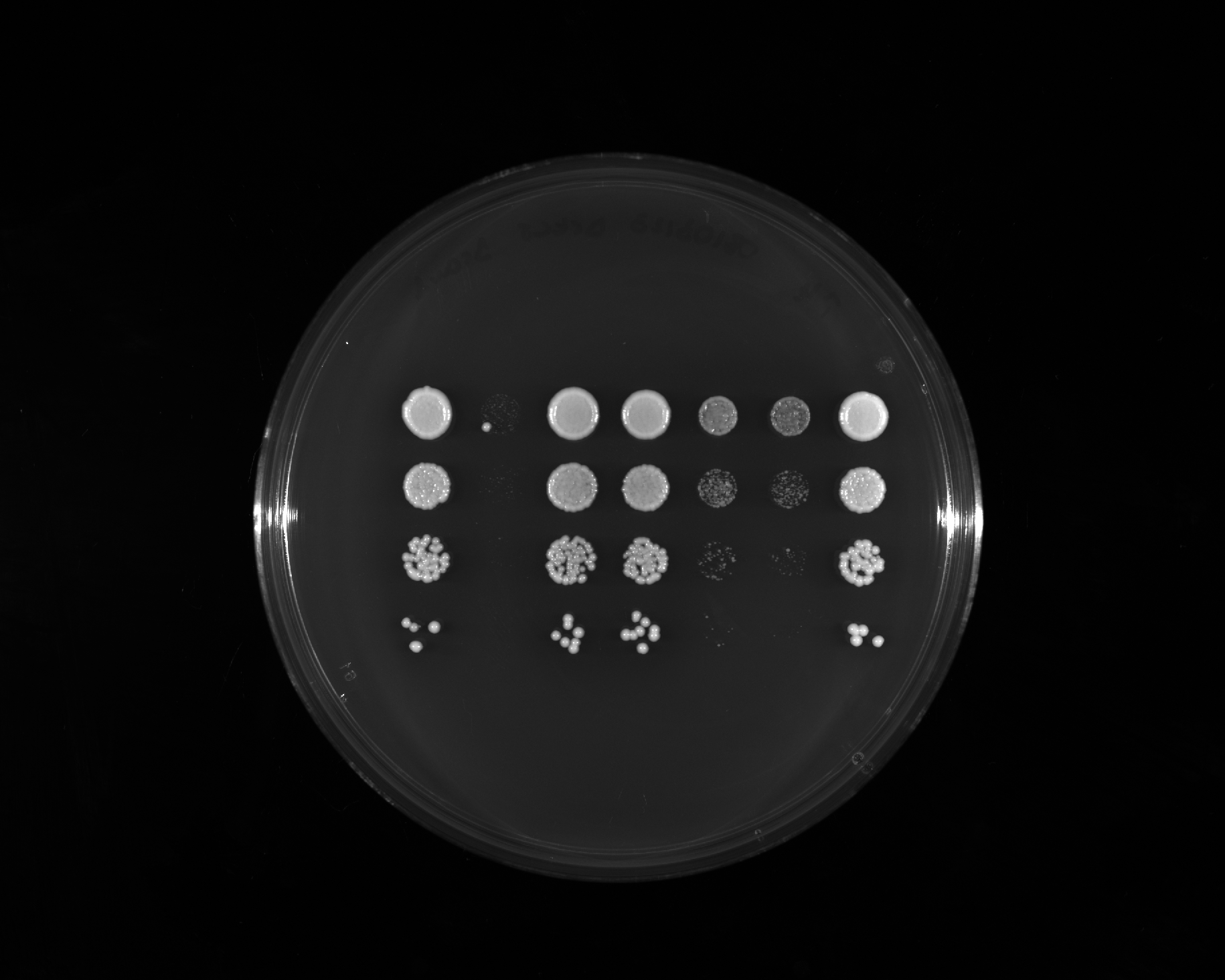

Supplement: Supplementary file 12 — Source data Fig. 7 [file 44318_2024_97_MOESM12_ESM.zip › Figure 7/7F/YPAD pH=7.5 3 mM ZnCl2.tif]

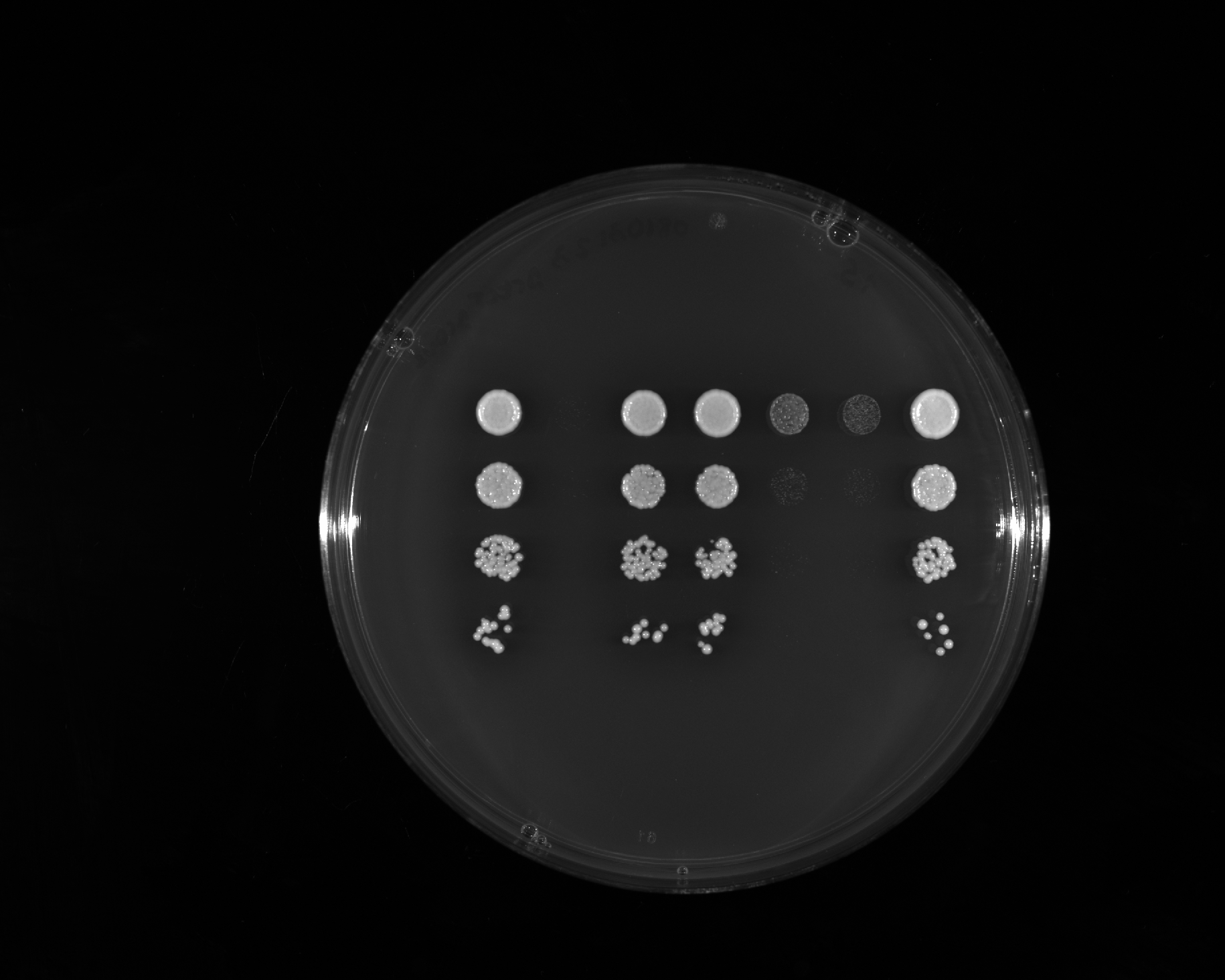

Supplement: Supplementary file 12 — Source data Fig. 7 [file 44318_2024_97_MOESM12_ESM.zip › Figure 7/7F/YPAD pH=7.5 6 mM ZnCl2.tif]

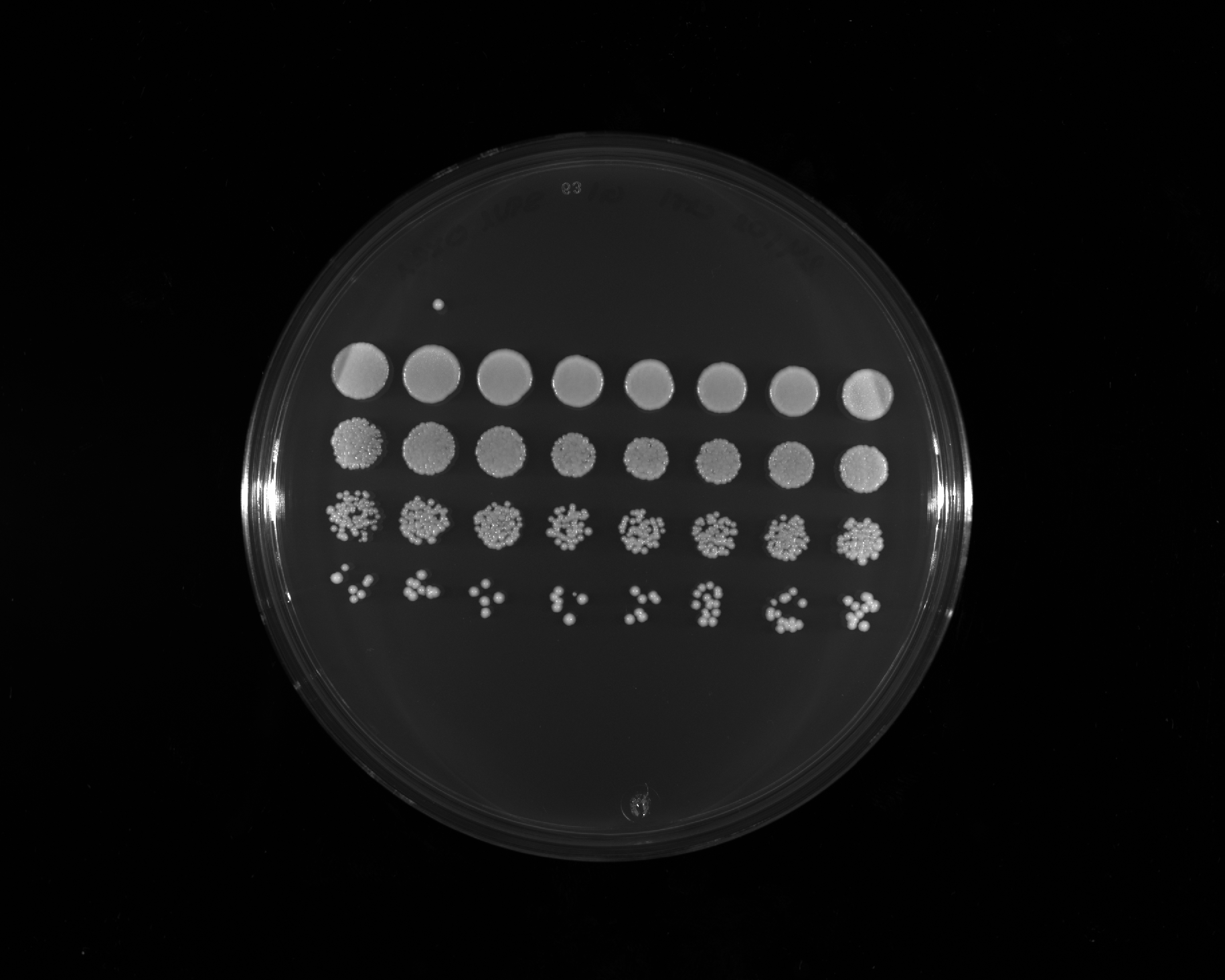

Supplement: Supplementary file 12 — Source data Fig. 7 [file 44318_2024_97_MOESM12_ESM.zip › Figure 7/7H/YPAD pH=5.5.tif]

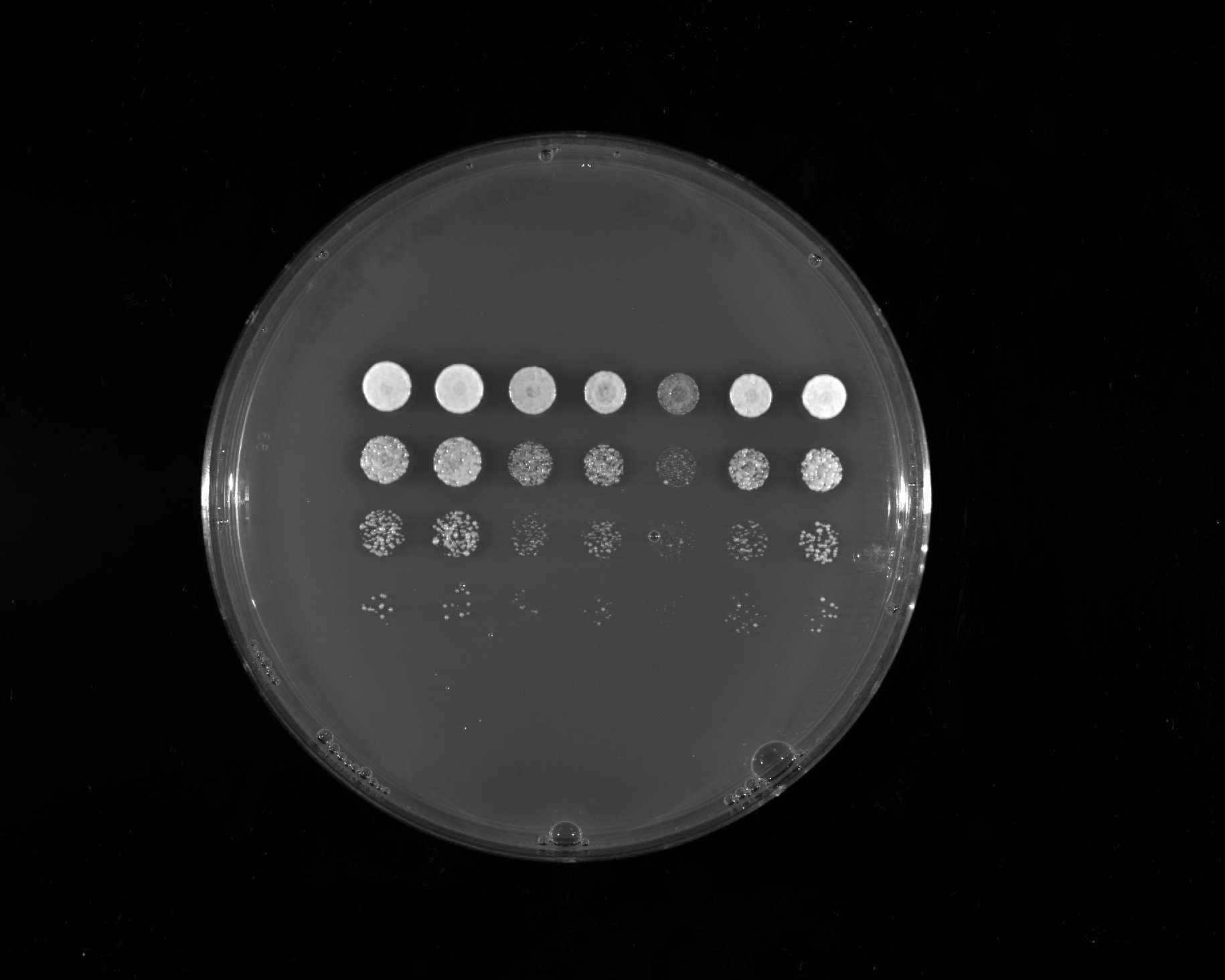

Supplement: Supplementary file 12 — Source data Fig. 7 [file 44318_2024_97_MOESM12_ESM.zip › Figure 7/7H/YPAD pH=7.5 6 mM ZnCl2.tif]

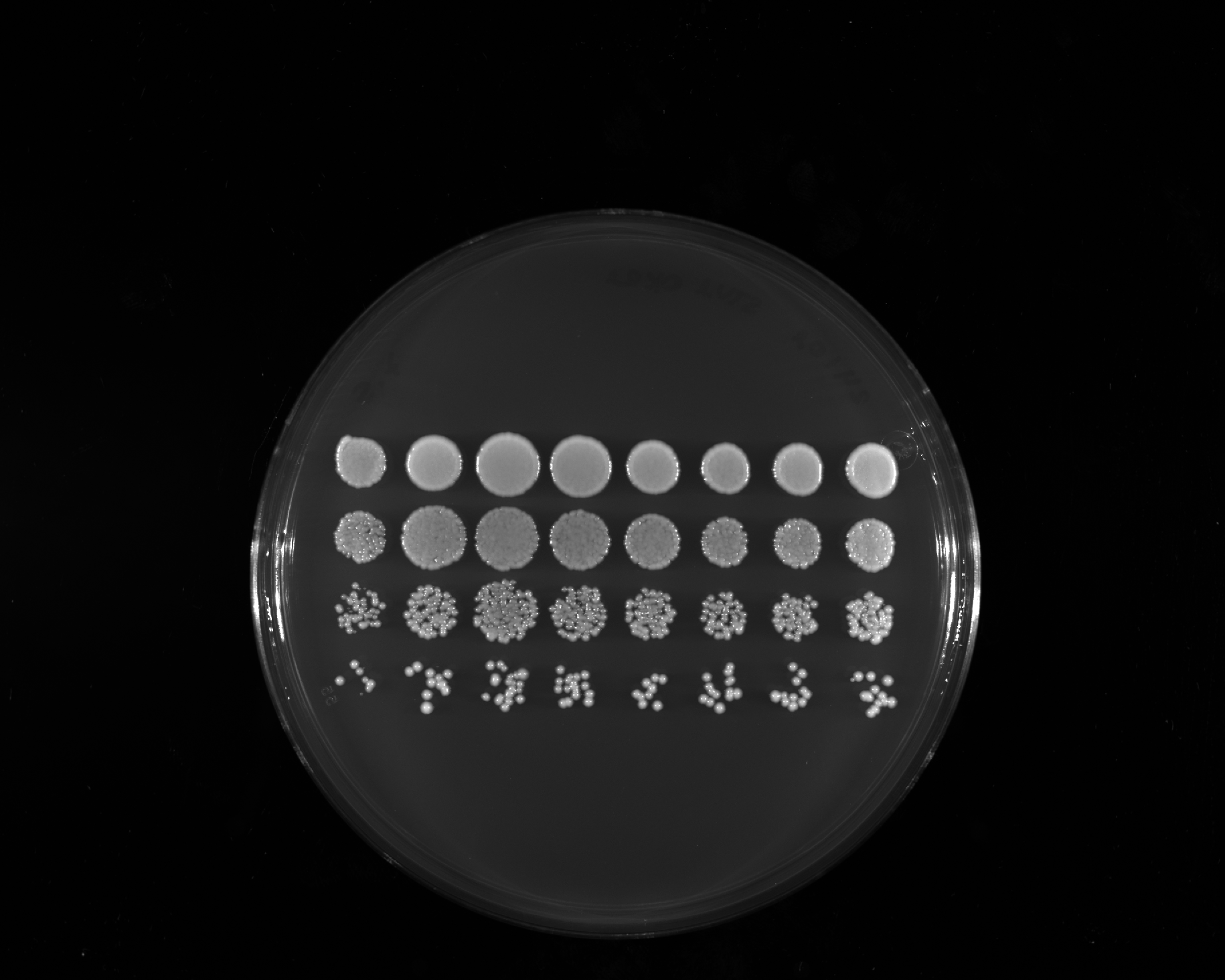

Supplement: Supplementary file 12 — Source data Fig. 7 [file 44318_2024_97_MOESM12_ESM.zip › Figure 7/7H/YPAD pH=7.5.tif]

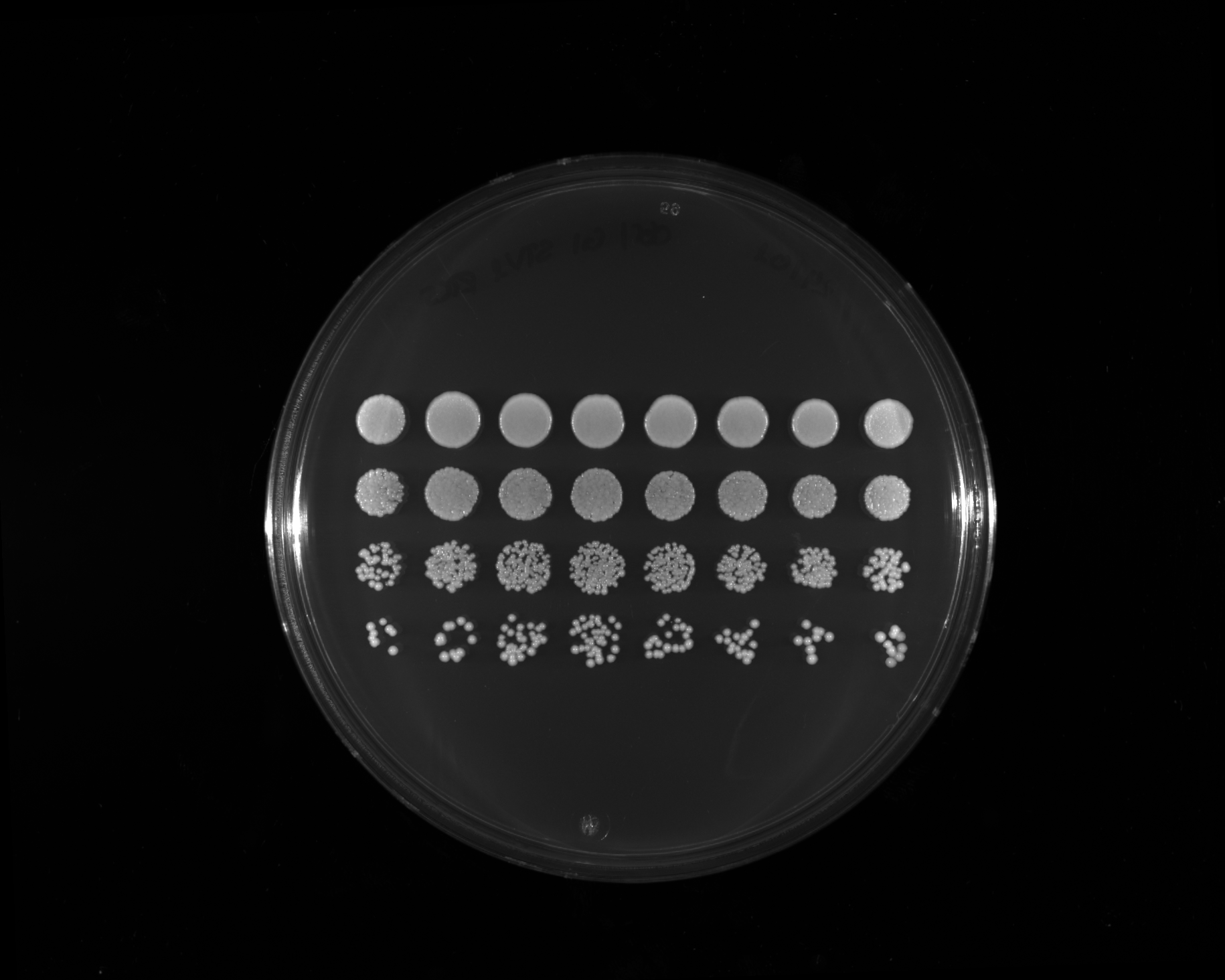

Supplement: Supplementary file 12 — Source data Fig. 7 [file 44318_2024_97_MOESM12_ESM.zip › Figure 7/7I/YPAD pH=5.5.tif]

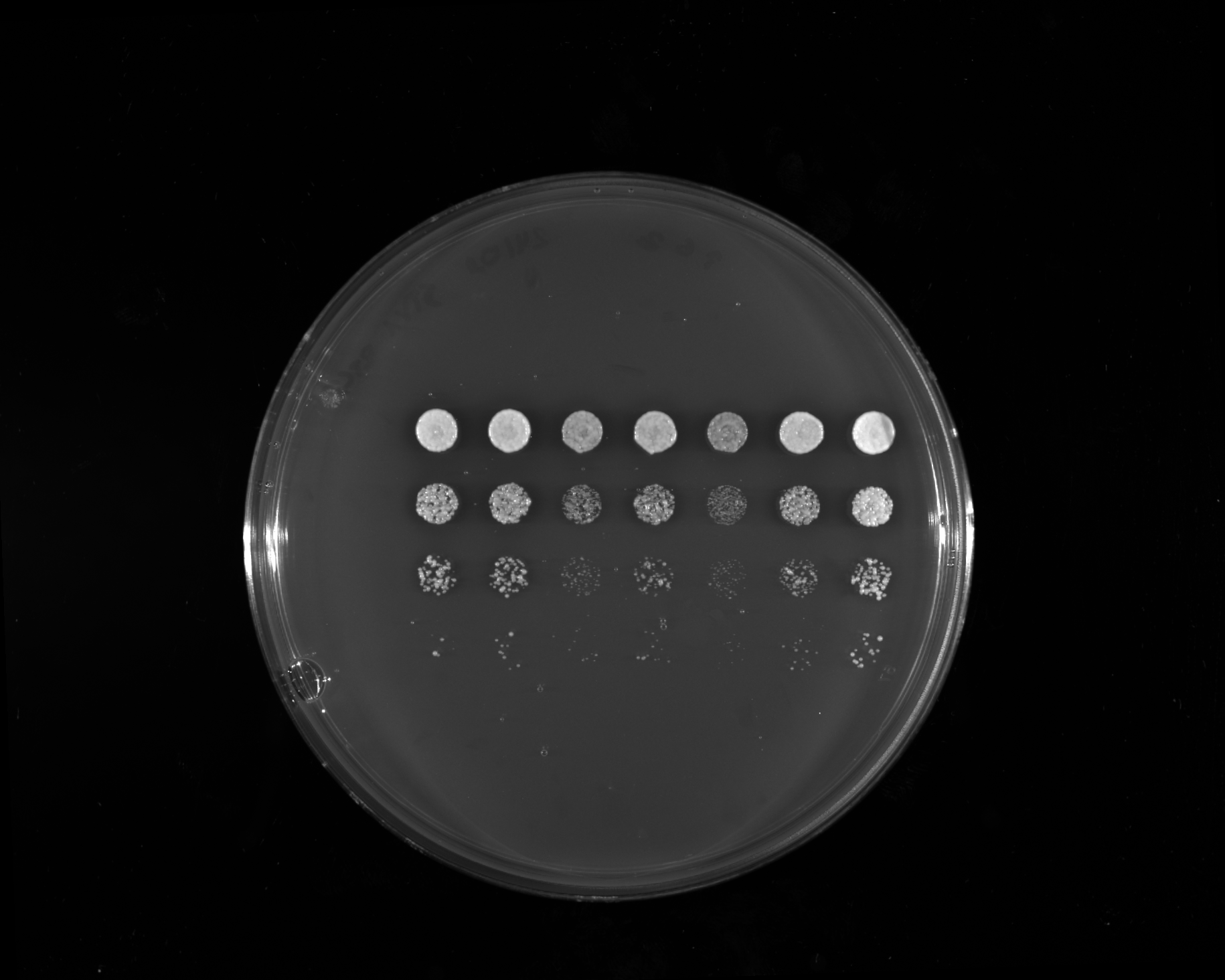

Supplement: Supplementary file 12 — Source data Fig. 7 [file 44318_2024_97_MOESM12_ESM.zip › Figure 7/7I/YPAD pH=7.5 6 mM ZnCl2.tif]

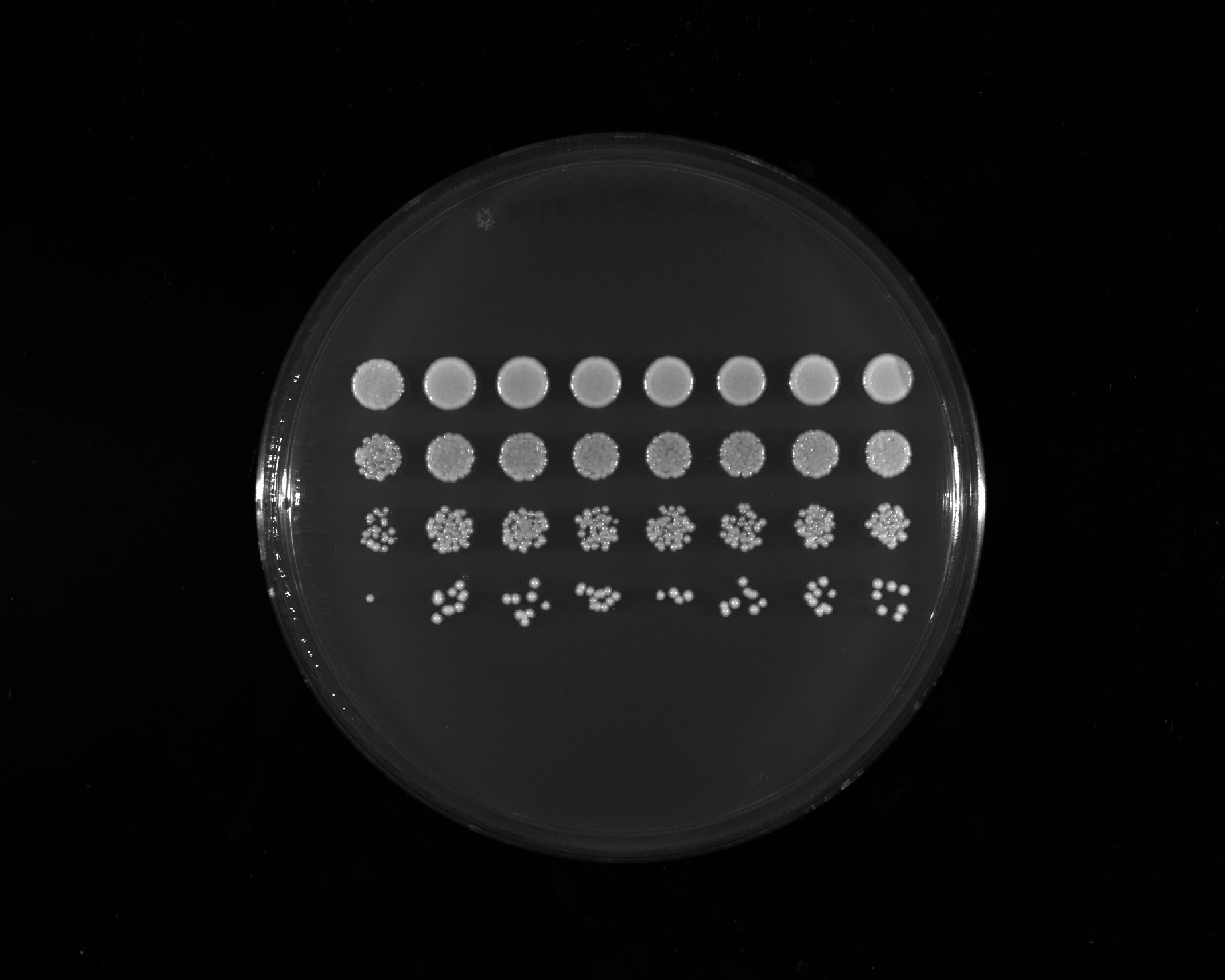

Supplement: Supplementary file 12 — Source data Fig. 7 [file 44318_2024_97_MOESM12_ESM.zip › Figure 7/7I/YPAD pH=7.5.tif]

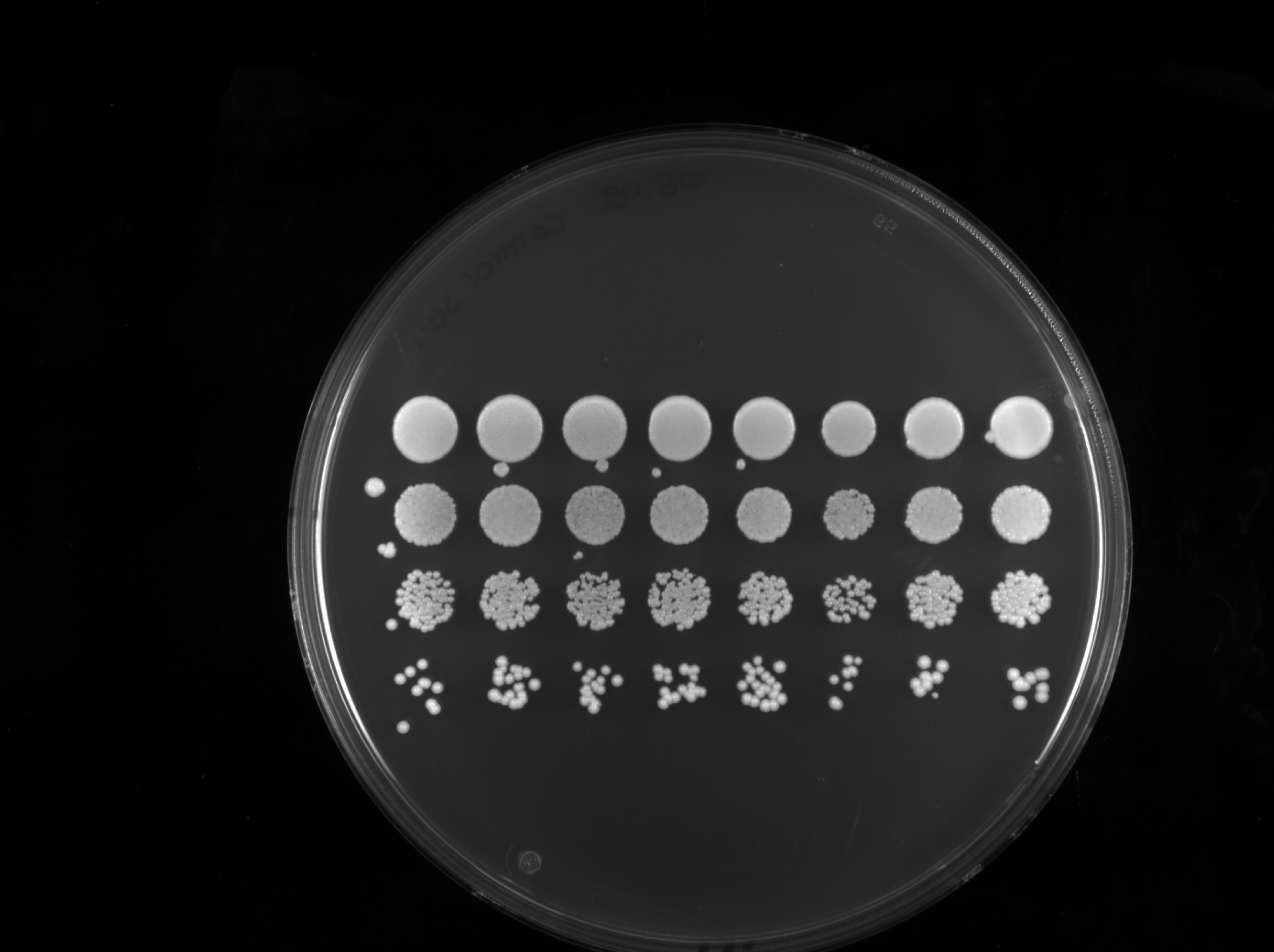

Supplement: Supplementary file 13 — Source data Fig. 8 [file 44318_2024_97_MOESM13_ESM.zip › Figure 8/8H/YPAD pH=5.5.tif]

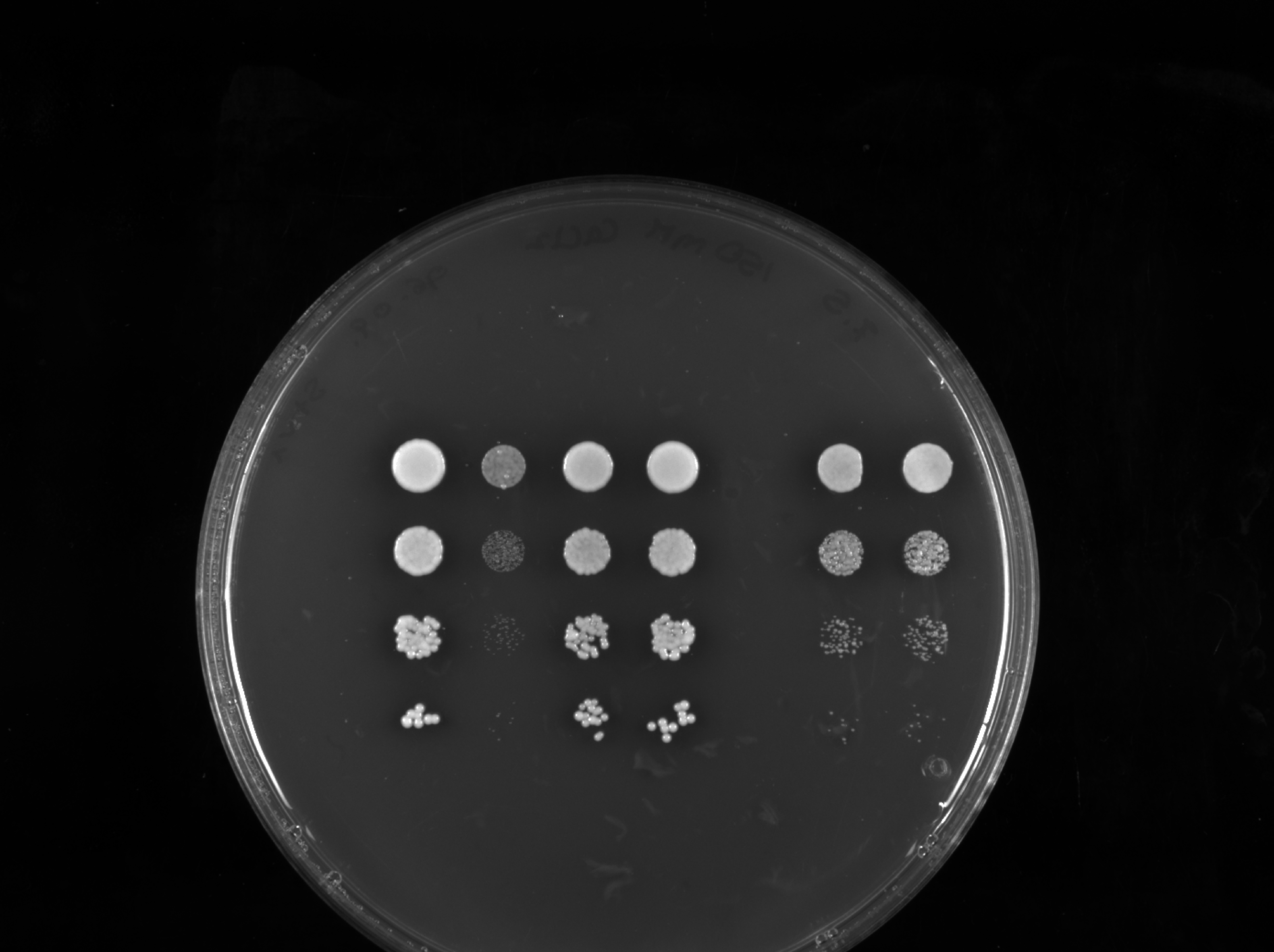

Supplement: Supplementary file 13 — Source data Fig. 8 [file 44318_2024_97_MOESM13_ESM.zip › Figure 8/8H/YPAD pH=7.5 150 mM CaCl2.tif]

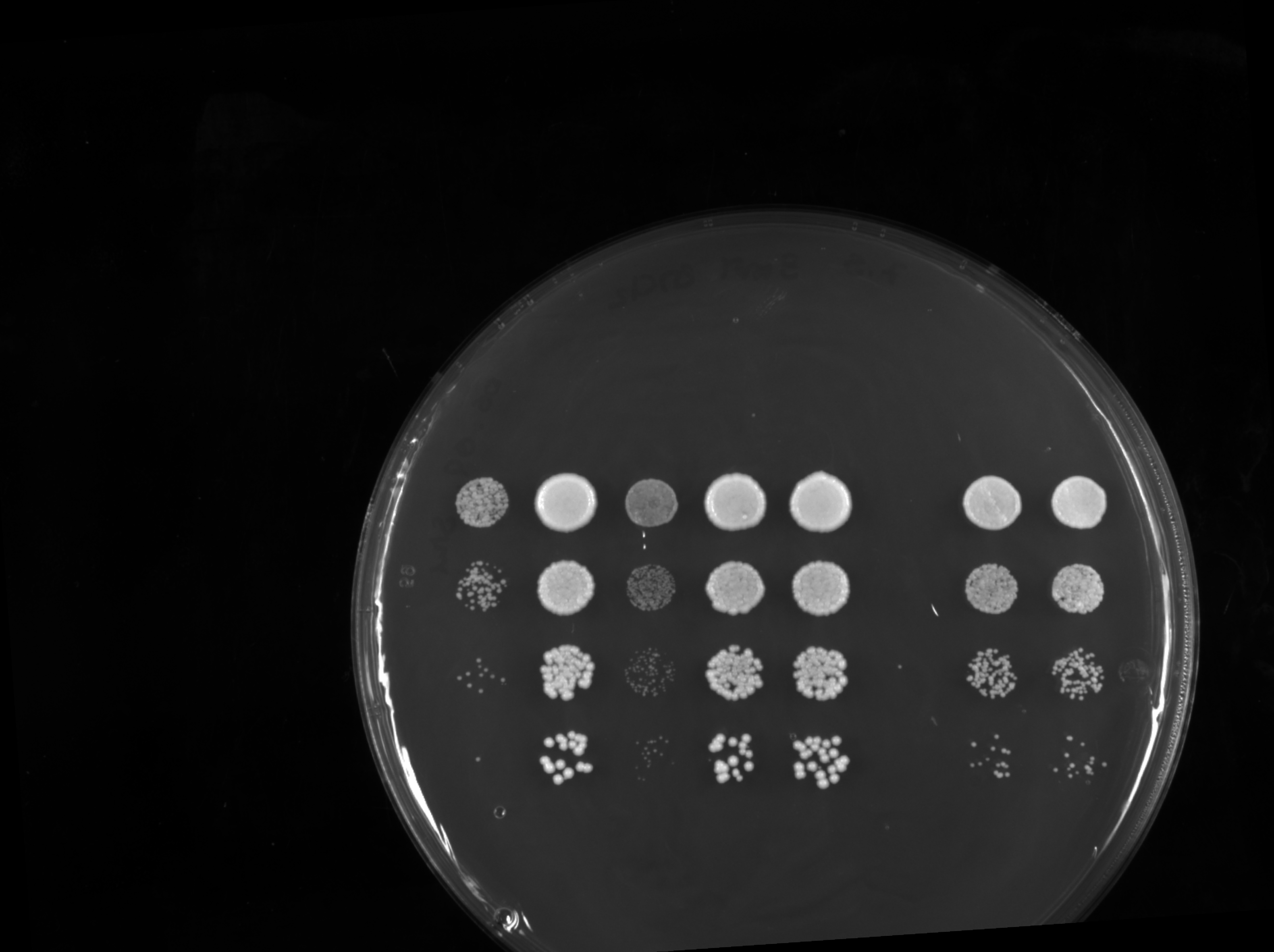

Supplement: Supplementary file 13 — Source data Fig. 8 [file 44318_2024_97_MOESM13_ESM.zip › Figure 8/8H/YPAD pH=7.5 3 mM ZnCl2.tif]
