# Supplementary material for: Yeast TLDc domain proteins regulate assembly state and subcellular localization of the V-ATPase
Source: EMBO J. 2024 Apr 8;43(9):9. doi: 10.1038/s44318-024-00097-2 (PMC11066047; doi:10.1038/s44318-024-00097-2)
Supplement: Supplementary file 15 — Expanded View Figures [file 44318_2024_97_MOESM15_ESM.pdf]

Expanded View Figures

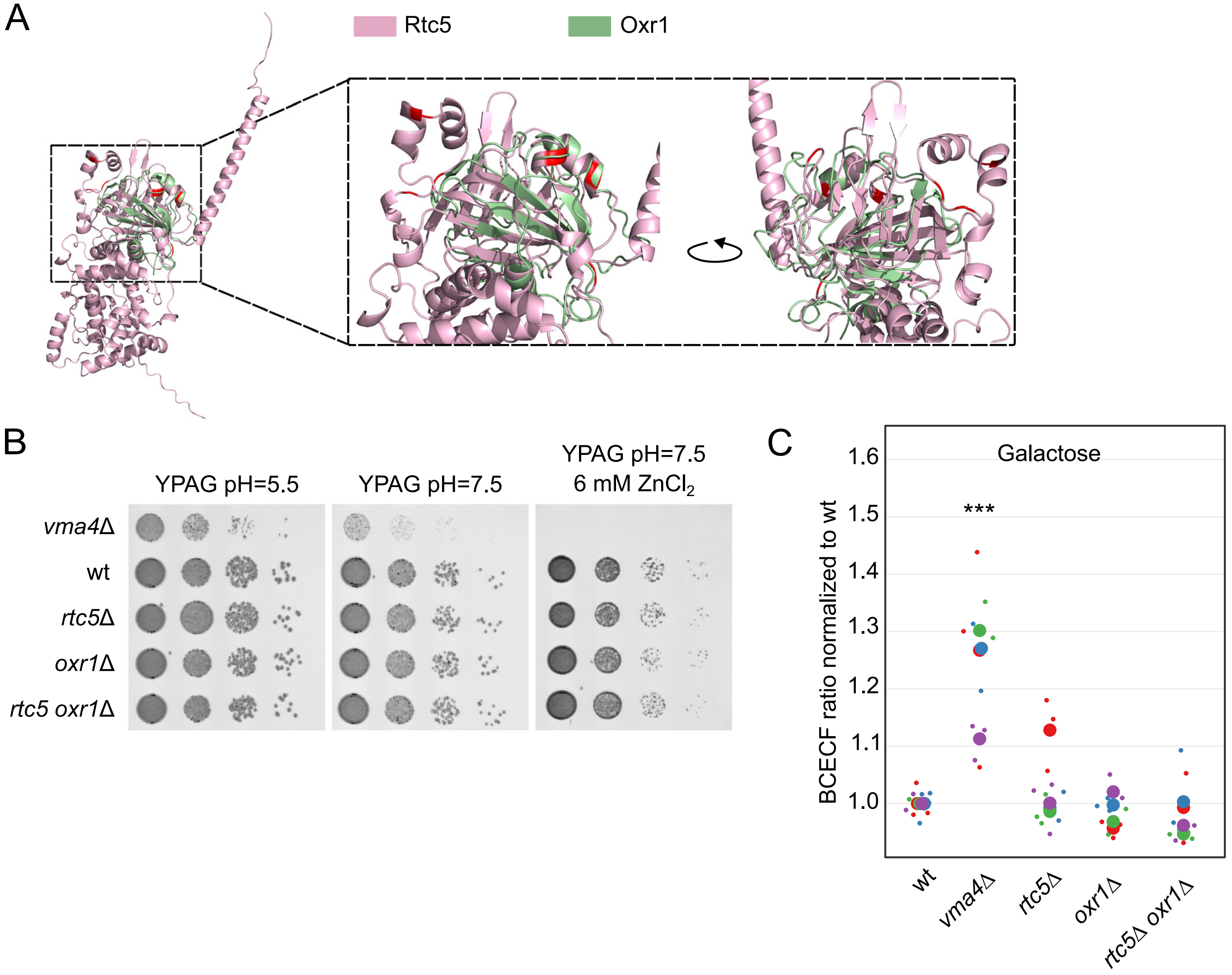

Figure EV1. Alphafold model of Rtc5 and growth phenotypes of *rtc5Δ* and *oxr1Δ* strains in medium containing galactose as the carbon source.

(**A**) Comparison of the AlphaFold model generated for Rtc5 with the available structure of Oxr1, the only two TLDc domain-containing proteins of *Saccharomyces cerevisiae*. (**B**) A wt strain or strains lacking *VMA4*, *OXR1* or *RTC5*, or both *OXR1* and *RTC5* were spotted as serial dilutions on media containing galactose as the carbon source with pH = 5.5, pH = 7.5, or pH = 7.5 and 6 mM $ZnCl_2$. (**C**) Analysis of vacuolar acidity via BCECF staining in a wt strain, a strain lacking *VMA4, OXR1, RTC5*, or both *RTC5* and *OXR1*. The experiments were performed with cultures grown in a medium containing galactose and pH = 5.5. For each strain, at least three independent experiments were performed, each containing three biological replicates. For each sample, the fluorescence emission of BCECF at 538 nm was measured when excited at 440 or 485 nm, and a ratio between these two values was calculated. The ratio was normalized to the average value for the wt strain in that experiment. The different colors in the graph indicate independent experiments, the smaller dots are biological replicates and the larger circles represent the averages of each independent experiment. Statistical analysis was performed with a one-way ANOVA and a Tukey post hoc test. The *vma4Δ* strain was significantly different from the wt strain (****P* value <0.001), all other strains are not significantly different from the wt strain (*P* value >0.05).

 © The Author(s)

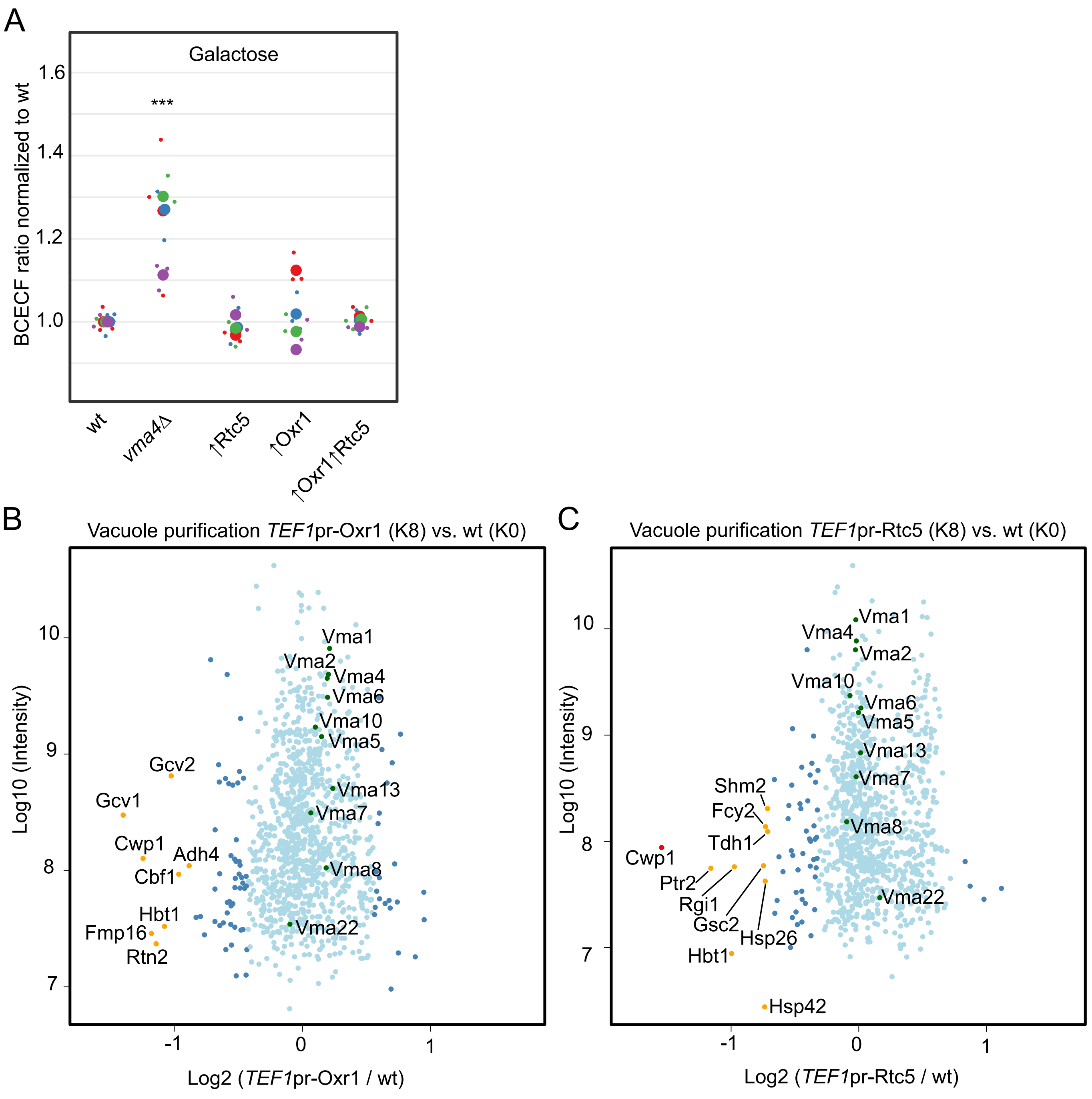

Figure EV2. Vacuolar acidity when grown in galactose and vacuolar proteomics of strains overexpressing Rtc5 or Oxr1.

(**A**) Analysis of vacuolar acidity via BCECF staining in a wt strain, a strain lacking *VMA4*, overexpressing Oxr1, Rtc5, or both Rtc5 and Oxr1. The experiments were performed with cultures grown in a medium containing galactose and pH = 5.5. Four independent experiments were performed, each containing three biological replicates. For each sample, the fluorescence emission of BCECF at 538 nm was measured when excited at 440 or 485 nm, and a ratio between these two values was calculated. The ratio was normalized to the average value for the wt strain in that experiment. The different colors in the graph indicate independent experiments, the smaller dots are biological replicates, and the larger circles represent the averages of each independent experiment. Statistical analysis was performed with a one-way ANOVA and a Tukey post hoc test. The *vma4*Δ strain was significantly different from the wt strain (****P* value <0.001), all other strains are not significantly different from the wt strain (*P* value >0.05). (**B**, **C**) SILAC-based vacuole proteomics of cells overexpressing either Oxr1 (**B**) or Rtc5 (**C**) compared with the wt strain. Log10 of the detected protein intensities are plotted against Log2 of the heavy/light SILAC ratios. Significant outliers are color-coded in red (*P* <1e − 14), orange (*P* < 0.0001), or dark blue (*P* < 0.05); other identified proteins are shown in light blue. V-ATPase subunits are labeled and shown as green dots. Statistical comparison is based on a two-group, two-tailed Student´s *t*-test. In panel **C** the range chosen for the X-axis excludes the dot representing Rtc5, so that the individual dots are clearly visible. This protein showed a Log2 (normalized H/L ratio) of 4.109945 and a Log10 (intensity) 9.433689846.

© The Author(s)

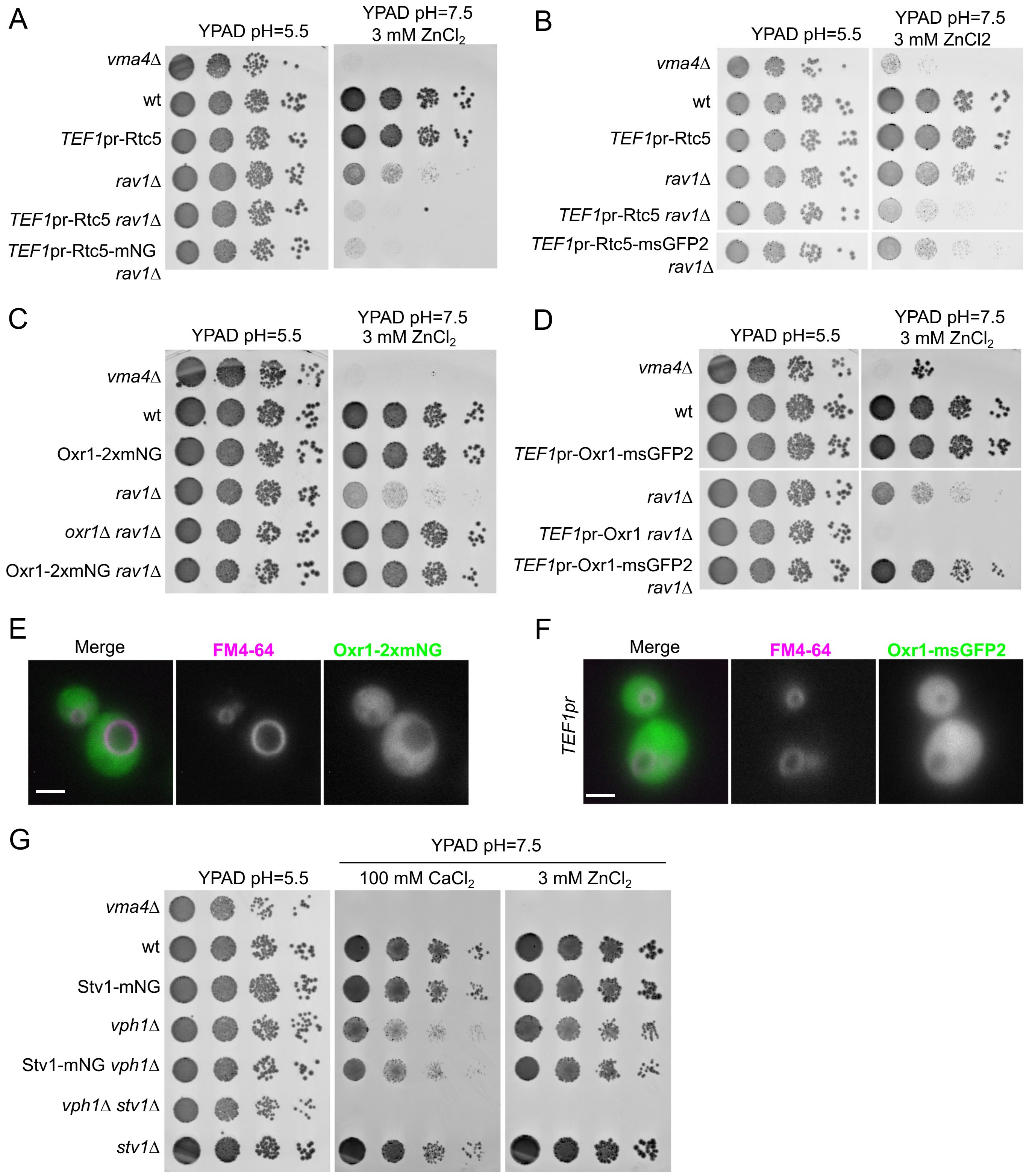
A
YPAD pH=5.5
YPAD pH=7.5 3 mM ZnCl2
vma4Δ
wt
TEF1pr-Rtc5
rav1Δ
TEF1pr-Rtc5 rav1Δ
TEF1pr-Rtc5-mNG rav1Δ
B
YPAD pH=5.5
YPAD pH=7.5 3 mM ZnCl2
vma4Δ
wt
TEF1pr-Rtc5
rav1Δ
TEF1pr-Rtc5 rav1Δ
TEF1pr-Rtc5-msGFP2 rav1Δ
C
YPAD pH=5.5
YPAD pH=7.5 3 mM ZnCl2
vma4Δ
wt
Oxr1-2xmNG
rav1Δ
oxr1Δ rav1Δ
Oxr1-2xmNG rav1Δ
D
YPAD pH=5.5
YPAD pH=7.5 3 mM ZnCl2
vma4Δ
wt
TEF1pr-Oxr1-msGFP2
rav1Δ
TEF1pr-Oxr1 rav1Δ
TEF1pr-Oxr1-msGFP2 rav1Δ
E
Merge
FM4-64
Oxr1-2xmNG
F
Merge
FM4-64
Oxr1-msGFP2
TEF1pr
G
YPAD pH=5.5
YPAD pH=7.5
100 mM CaCl2
3 mM ZnCl2
vma4Δ
wt
Stv1-mNG
vph1Δ
Stv1-mNG vph1Δ
vph1Δ stv1Δ
stv1Δ

 © The Author(s)

Figure EV3. C-terminally tagged Rtc5 and Stv1 are functional, C-terminally tagged Oxr1 is not.

(**A**, **B**) Rct5-mNeonGreen and Rtc5-msGFP2 are functional. Serial dilutions of strains with the indicated genotypes were spotted on YPAD media pH = 5.5 or YPAD media pH = 7.5 containing 3 mM $ZnCl_2$. (**C**) Oxr1-2xmNeonGreen is not functional. Serial dilutions of strains with the indicated genotypes were spotted on YPAD media pH = 5.5 or YPAD media pH = 7.5 containing 3 mM $ZnCl_2$. (**D**) Oxr1-msGFP2 is not functional. Serial dilutions of strains with the indicated genotypes were spotted on YPAD media pH = 5.5 or YPAD media pH = 7.5 containing 3 mM $ZnCl_2$. (**E**) Oxr1-2xmNeonGreen shows a cytosolic localization. Fluorescence microscopy images of cells expressing Oxr1-2xmNeonGreen (2xmNG) and endocytosed FM4-64 as a vacuolar marker. The scale bar represents 2 µm. (**F**) Overexpressed Oxr1-msGFP2 shows a cytosolic localization. Fluorescence microscopy images of cells expressing Oxr1-msGFP2 under the control of the strong constitutive *TEF1* promoter and endocytosed FM4-64 as a vacuolar marker. The scale bar represents 2 µm. (**G**) Stv1-mNeonGreen is functional. Strains with the indicated genotypes were spotted as seriated dilutions in YPAD medium pH=5.5 and YPAD medium pH = 7.5 containing either 100 mM $CaCl_2$ or 3 mM $ZnCl_2$.

© The Author(s)

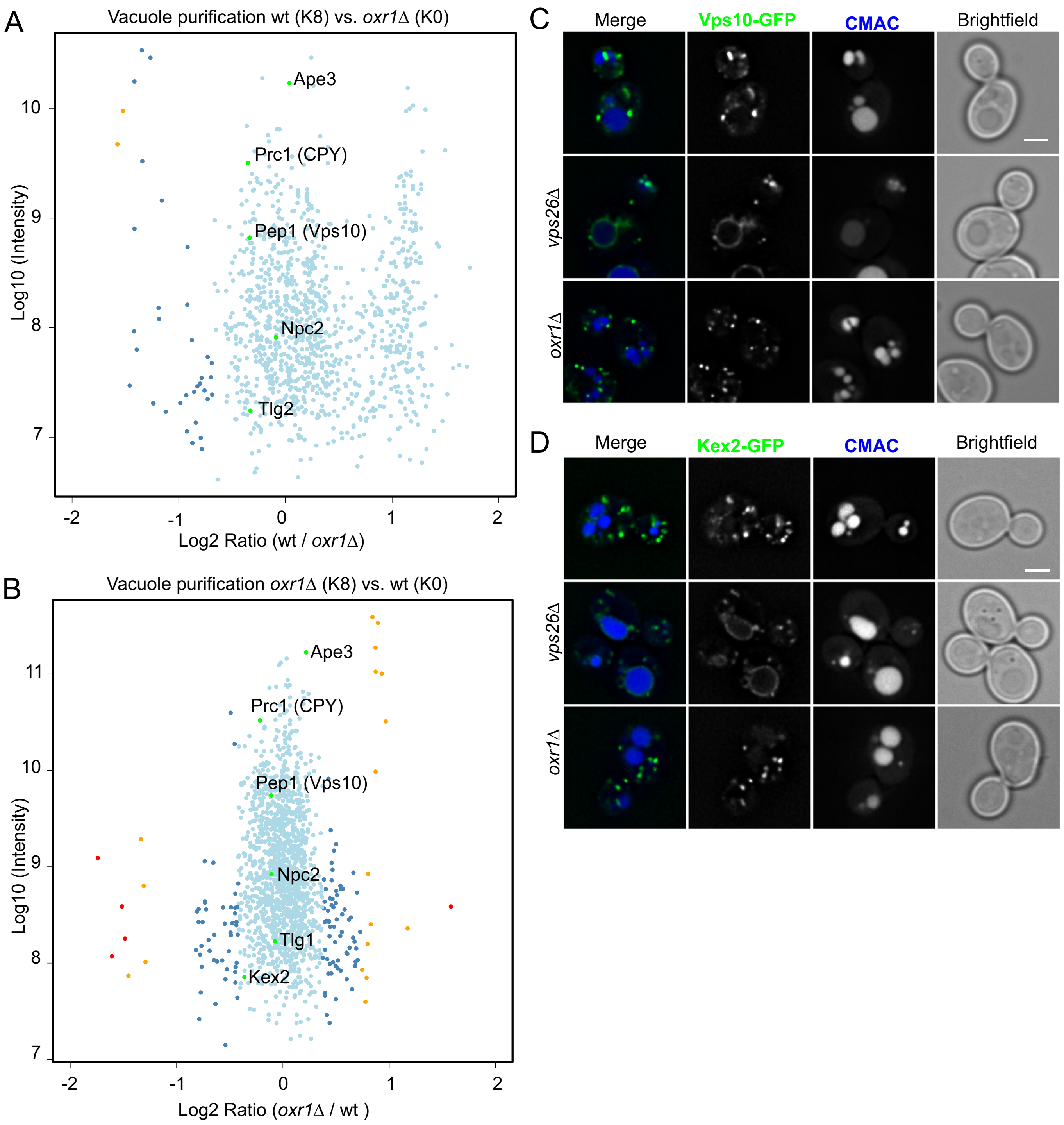

Figure EV4. The localization of cargo proteins of the Retromer pathway is not affected by deletion of *OXR1*.

(**A**, **B**) The abundance of Retromer cargo proteins in the vacuole is not affected by the deletion of *OXR1*. The experiment in (**A**) is the same experiment as in Appendix Fig. S3A and the experiment in panel (**B**) is the same experiment as the one in Fig. 5D. SILAC-based vacuole proteomics of cells lacking *OXR1* compared with the wt strain. Log10 of the detected protein intensities are plotted against Log2 of the heavy/light SILAC ratios. Significant outliers are color-coded in red (P value <1e − 14), orange (P value <0.0001), or dark blue (P value <0.05); other identified proteins are shown in light blue. Statistical comparison is based on a two-group, two-tailed Student´s t-test. Retromer cargo proteins were labeled and the dots are shown in green. (**C**, **D**) Retromer cargo proteins do not re-localize to the vacuole in strains lacking *OXR1*. Fluorescence microscopy analysis of Vps10-GFP or Kex2-GFP and vacuole lumen stained with CMAC, in wt cells, cells lacking the Retromer complex subunit *VPS26* or strains lacking *OXR1*. The scale bar represents 2 μm.

 © The Author(s)
